# Chromosome-Scale Genome of Masked Palm Civet (*Paguma larvata*) Shows Genomic Signatures of Its Biological Characteristics and Evolution

**DOI:** 10.3389/fgene.2021.819493

**Published:** 2022-01-21

**Authors:** Tianming Lan, Dongming Fang, Haimeng Li, Sunil Kumar Sahu, Qing Wang, Hao Yuan, Yixin Zhu, Zipeng Yang, Le Zhang, Shangchen Yang, Haorong Lu, Lei Han, Shaofang Zhang, Jieyao Yu, Yasser S. Mahmmod, Yanchun Xu, Yan Hua, Fengping He, Ziguo Yuan, Huan Liu

**Affiliations:** ^1^ State Key Laboratory of Agricultural Genomics, BGI-Shenzhen, Shenzhen, China; ^2^ College of Life Sciences, University of Chinese Academy of Sciences, Beijing, China; ^3^ College of Veterinary Medicine, South China Agricultural University, Guangzhou, China; ^4^ Key Laboratory of Zoonosis Prevention and Control of Guangdong Province, Guangzhou, China; ^5^ College of Wildlife and Protected Area, Northeast Forestry University, Harbin, China; ^6^ College of Life Sciences, Zhejiang University, Hangzhou, China; ^7^ China National GeneBank, BGI-Shenzhen, Shenzhen, China; ^8^ Guangdong Provincial Key Laboratory of Genome Read and Write, BGI-Shenzhen, Shenzhen, China; ^9^ Department of Veterinary Sciences, Faculty of Health Sciences, Higher Colleges of Technology, Al Ain, United Arab Emirates; ^10^ Division of Infectious Diseases, Department of Animal Medicine, Faculty of Veterinary Medicine, Zagazig University, Zagazig, Egypt; ^11^ Guangdong Provincial Key Laboratory of Silviculture, Protection and Utilization, Guangdong Academy of Forestry, Guangzhou, China; ^12^ College of Veterinary Medicine, Yunnan Agricultural University, Kunming, China

**Keywords:** masked palm civet, genome assembly, omnivorous diet, immune system, SARS-CoV-2, genetic diversity

## Abstract

The masked palm civet (*Paguma larvata*) is a small carnivore with distinct biological characteristics, that likes an omnivorous diet and also serves as a vector of pathogens. Although this species is not an endangered animal, its population is reportedly declining. Since the severe acute respiratory syndrome (SARS) epidemic in 2003, the public has been particularly concerned about this species. Here, we present the first genome of the *P. larvata*, comprising 22 chromosomes assembled using single-tube long fragment read (stLFR) and Hi-C technologies. The genome length is 2.41 Gb with a scaffold N50 of 105.6 Mb. We identified the 107.13 Mb X chromosome and one 1.34 Mb Y-linked scaffold and validated them by resequencing 45 *P. larvata* individuals. We predicted 18,340 protein-coding genes, among which 18,333 genes were functionally annotated. Interestingly, several biological pathways related to immune defenses were found to be significantly expanded. Also, more than 40% of the enriched pathways on the positively selected genes (PSGs) were identified to be closely related to immunity and survival. These enriched gene families were inferred to be essential for the *P. larvata* for defense against the pathogens. However, we did not find a direct genomic basis for its adaptation to omnivorous diet despite multiple attempts of comparative genomic analysis. In addition, we evaluated the susceptibility of the *P. larvata* to the SARS-CoV-2 by screening the RNA expression of the *ACE2* and *TMPRSS2/TMPRSS4* genes in 16 organs. Finally, we explored the genome-wide heterozygosity and compared it with other animals to evaluate the population status of this species. Taken together, this chromosome-scale genome of the *P. larvata* provides a necessary resource and insights for understanding the genetic basis of its biological characteristics, evolution, and disease transmission control.

## Introduction

The masked palm civet (*Paguma larvata*) (Carnivora: Viverridae) attracted public concern in 2003 when the severe acute respiratory syndrome (SARS)-associated coronavirus (SARS-CoV) was identified by Guan et al. from several *P. larvata* individuals from a Shenzhen (China) market (Guan et al., 2003). This small carnivore is the only species in the genus of *Paguma*. The distribution of this species is mainly restricted in subtropical and tropical areas of Asia (Jennings and Veron, 2009; Torii, 2009), but it can also be found in several northern provinces of China, such as Shanxi, Shaanxi, and Tibet (Gao, 1987; Wang, 2003; Smith and Xie, 2008). The masked palm civet was found to hibernate slightly during the winter in the northern area (Zhang et al., 1991; Kang et al., 1997). Besides, it has been introduced in Japan as an alien species (Nawa, 1965; Torii, 2009). This species is nocturnal, arboreal, and basically solitary. They live in a variety of habitats, including forests, rainforest, parks, fruit orchards, and gardens. They choose habitats based on the availability of food. There are 15 subspecies according to body color and facial pattern (Wozencraft, 2005), but the genetic difference among different populations is low (Patou et al., 2009), indicating the need for taxonomic revision for this species.

When compared to just maximizing energy gain, the most profitable forging approach for predators is to maximize the trade-off between forging costs and energetic rewards (Stein, 1977). Factors such as prey size and nutritions usually affect this trade-off over space and time (Hörnfeldt, 1978; Sundell et al., 2003). The *P. larvata* is a dietary generalist and consumes a broad spectrum of prey items that primarily comprise small mammals and fruits, including frogs, snakes, birds, and even invertebrates, plant cortexes, and leaves (Zhou et al., 2008; Iwama et al., 2017). From June to October, the highest consumption of the *P. larvata* is fruits largely due to the abundance of fruits in this period (Song and Liu, 1999; Zhou et al., 2008), but their main diet will switch to small mammals when fruits are at its lowest abundance. In addition, birds will become the main food of the *P. larvata* in spring and winter, possibly because birds are easier to be caught due to loss of leaves on the trees (Wang et al., 1997; Song and Liu, 1999; Zhou et al., 2008). Besides, diets between female and male are also different; for instance, insects and amphibians are more frequently consumed by females, likely related to raising the offspring (Iwama et al., 2017). But the genetic basis for such a general diet is largely unknown and needs further exploration.

The masked palm civet is also an important vector of viruses, parasites, and bacteria, and related diseases can be also zoonotically transmitted to humans (Shi and Hu, 2008; Lee et al., 2011; Sato et al., 2013; Hou et al., 2016; Wicker et al., 2017; Yu et al., 2020), posing a great threat to public health. With the outbreak of SARS in 2003, several studies have confirmed that SARS-CoV-like viruses can be identified in *P. larvata* individuals (Shi and Hu, 2008), raising an alert for SARS-CoV transmission by the *P. larvata.* In addition, other zoonotic pathogens were also reported to be carried and transmitted by the *P. larvata*, including *Toxoplasma gondii*, *Enterocytozoon bieneusi*, *Bartonella henselae*, *Giardia duodenalis*, *Salmonella enterica*, *Campylobacter* spp., and *Cryptosporidium* spp. (Lee et al., 2011; Sato et al., 2013; Hou et al., 2016; Wicker et al., 2017; Yu et al., 2020). The *P. larvata* becomes the most potential carrier and transmitter for all diseases related with the above mentioned pathogens, especially under the situation of being asymptomatic after being affected by these pathogens. Therefore, it is seriously necessary to screen the most possibly susceptible tissues of the *P. larvata* for different pathogens to support the prevention of pathogen transmission. Also, how the *P. larvata* develops mechanisms to resist pathogens is vague and still needs further close investigation.

In this study, we present the first genome assembly of the *P. larvata* individual. We explored the possible genetic basis of their omnivorous diet as a carnivore and of their ability to carry multiple pathogens without serious symptoms. We also predicted the most susceptible organs of the *P. larvata* to be infected by the SARS-CoV-2 by screening the gene expression of *ACE2* and *TMPRSS2*/*TMPRSS4* genes in 16 organs, contributing to the management of the transmission of the SARS-CoV-2 between humans and animals.

## Materials and Methods

### Samples and Ethics Statement

A male *P. larvata* individual from Guangdong Provincial Wildlife Rescue Center was collected for genome assembly and RNA sequencing. This individual died of natural causes and was immediately stored in liquid nitrogen after a quick dissection. We isolated 16 tissues/organs for RNA sequencing, including the heart, lung, spleen, liver, kidney, esophagus, stomach, colon, rectum, cecum, duodenum, jejunum, testis, vas deferens, bladder, and spinal cord. The muscle sample was used for single-tube long fragment read (stLFR) sequencing and genome survey. The liver sample was used for Hi-C sequencing. We also collected muscle samples from another 45 individuals from Jiahe special animal breeding center in Guangdong for whole genome resequencing. Sample collection and research were both approved by the Institutional Review Board of BGI (BGI-IRB E21053). All procedures were conducted according to the guidelines from BGI-IRB.

### Nucleic Acid Isolation, Library Preparation, and Sequencing

Total RNA extraction was performed using TRlzol reagent (Invitrogen, United States) according to the manufacturer's instructions. RNA purity, integrity, and quantity were evaluated by Agilent 2,100 Bioanalyzer system (Agilent, United States) and Qubit 3.0 (Life Technologies, United States). The isolated RNA was fragmented into 200–400 bp and reverse-transcribed to cDNA for library preparation. High-molecular-weight DNA was isolated by using the protocol described by Wang et al. (2019). We constructed two stLFR co-barcoding DNA libraries using the MGIEasy stLFR Library Prep Kit (MGI, China). Qiagen Blood and Cell Culture DNA Mini Kit (Qiagen, United States) was used for genomic DNA extraction. One Hi-C library was prepared with the restriction endonuclease *dpnII*. Ninety-four short-insert-size (∼250 bp) libraries (48 for cDNA, 46 for genomic DNA) were finally constructed according to the manufacture's instruction. All the 97 libraries were finally subject to the DNBSEQ-T1 sequencer (MGI, China) for 100-bp paired-end sequencing.

### Genome Assembly, Annotation, and Assessment

Here, the *P. larvata* genome size was estimated by using K-mer frequencies, according to the Lander–Waterman theory (Lander and Waterman, 1988). Supernova (v2.1.1) (Weisenfeld et al., 2017) was used for the assembly of the primary genome by using stLFR sequencing data with default parameters. GapCloser (Luo et al., 2012) and purge_dups (v1.2.3) were then used for filling gaps and redundancy removal. Finally, we used 3d-DNA pipeline (v180922) (Durand et al., 2016) to concatenate the stLFR assembled scaffolds to the chromosome-scale genome. Protein-coding genes were inferred using *de novo*, homology-based, and RNA-seq approaches. *De novo* gene prediction was performed on a repeat-masked genome using Augustus (v3.0.3) (Stanke et al., 2004), GlimmerHMM (v3.0.1) (Majoros et al., 2004), and SNAP (v11/29/2013) (Korf, 2004). Training models were generated from a subset of the transcriptomic data representing 800 distinct genes. Homologous gene prediction was performed by comparing protein sequences of *Felis catus*, *Homo sapiens*, *Lynx canadensis*, *Mus musculus*, *Panthera pardus*, and uniprot database (release-2020_05). The final non-redundant gene set representing homology, *de novo*, and RNA-seq supported genes was generated using MAKER pipeline (v3.01.03) (Campbell et al., 2014). The completeness of the genome and gene set were evaluated by Benchmarking Universal Single-Copy Orthologs (BUSCO, v3.1.0) (Simão et al., 2015) analysis using the database of mammalia_odb9. For repeat identification, we firstly used the MITE-hunter (v4.07) (Han and Wessler, 2010), LTR finder (v1.0.6) (Xu and Wang, 2007) and RepeatModeler2 (v2.0.1) (Tarailo-Graovac and Chen, 2009; Flynn et al., 2020) to identify *de novo* repeat motifs. These repeats were then added into the RepBase as known elements to be subjected to RepeatMasker (v4.1.1) (Chen, 2004) to identify and classify transposable elements. Tandem Repeats Finder (v4.07) (Benson, 1999) was also used for searching tandem repeats across the genome.

### Gene Family and Orthologous Gene Identification

A comparative analysis was used to determine the relationship of homologous genes. Here we selected 20 species according to 1) the evolutionary relationship with the *P. larvata*, 2) the dietary characteristics, and 3) the quality of their genomes (Supplementary Table S1). First, the longest transcript of each gene from each species was used to perform all-to-all BLASTP analysis with the parameter “-evalue 1e−5.” Then, genes were clustered using Treefam (v1.4) (Li et al., 2006) with hierarchically clustering on a sparse graph. Finally, 20,830 gene families were identified in all 20 reference genomes, with 5,425 genes determined as single-copy genes shared by all these 20 species.

### Phylogeny Reconstruction and Divergence Time Estimation

Based on the 5,425 single-copy genes we identified, we constructed a phylogenetic tree that involved these 20 species. We first performed multiple amino acid sequence alignment by MAFFT (v.7.310) (Katoh and Standley, 2013) for each single-copy gene orthogroup. Then, the alignment of amino acid sequences was converted to an alignment file of DNA sequences using PAL2NAL (v14) (Suyama et al., 2006), followed by gap removal by the trimal (v1.4.1) software (Capella-Gutierrez et al., 2009). Finally, we built a phylogenetic tree based on the concatenated super-genes using IQTREE (v1.6.12) (Nguyen et al., 2015) with the maximum-likelihood (ML) algorithm. The best-fit substitution model was calculated using ModelFinder (Kalyaanamoorthy et al., 2017).

We used the MCMCTREE (v4.5) in the PAML software (Yang, 2007) to estimate the divergence time among species. The Markov chain Monte Carlo (MCMC) process was run for 1,500,000 iterations with a sampling frequency of 150 after a burn-in of 500,000 iterations. Convergence was checked by two independent runs. Sequences for 5,425 single-copy genes were used as the input file for MCMCTree, and multiple fossil time points were used for time calibrations from Timetree (http://www.timetree.org/).

### Analysis of Gene Expansion and Contraction

Based on the phylogenetic tree we constructed using the single-copy genes, we detected expanded and contracted gene families by the CAFE (v 4.2.1) (De Bie et al., 2006), with a random birth and death model, to estimate the size of each gene family at each ancestral node and obtain a family-wise *p*-value. Here, we explored expanded and contracted gene families in the *P. larvata* genome compared with all the above mentioned 19 species, 9 carnivores, 5 omnivores, and 5 herbivores, respectively. We performed the gene ontology (GO) and Kyoto Encyclopedia of Genes and Genomes (KEGG) enrichment analysis on these expanded gene families by setting the whole annotated gene set as the background. Fisher's exact test was used when expected gene counts were below 5 because this makes the chi-square test inaccurate. The computed *p*-value was adjusted for multiple tests by specifying a false discovery rate (q-value <0.05) using the Benjamini–Hochberg method (Benjamini and Yekutieli, 2005).

### Positively Selected Genes

Single-copy orthologs were extracted for positive selection analysis. We identified PSGs in the *P. larvata* genome compared with both all the 19 species and the 7 pure meat-eating Felidae animals, respectively. The branch-site model of the CodeML in the PAML (v4.8) (Yang, 2007) was used for calculating the ratio of nonsynonymous substitution per nonsynonymous site to synonymous substitution per synonymous site (dN/dS). Likelihood ratio tests (LTRs) were carried out to calculate the *p*-values by using chi-square statistics. The corrected *p*-value less than 0.05 was identified under positive selection.

### Phylogenetic Trees of Nine Gene Families

We first identify the nine candidate gene families, including CYP450, CES, GST, ABC, UGT, AOX, TAS2R, TAS1R, and AMY, to address concerns of spurious gene loss. We searched protein sequences from 19 species (Supplementary Table S1) against the *P. larvata* genome assembly using TBLASTN (v2.2.18) (Altschul et al., 1990), and homologous genes were predicted by GeneWise (v2.2.0) (Birney et al., 2004) and spaln (v2.4.4) (Iwata and Gotoh, 2012) with a high e-value threshold of 1e−3 and a low amino acid sequence similarity value of 0.3. After confirming the corresponding genes in the *P. larvata* genome, we constructed the ML gene tree for each of the nine gene families with the 19 species.

### Variants Calling and Quality Control

Firstly, resequencing data from 45 farmed adult masked palm civets were aligned to the *P. larvata* genome using the Burrows–Wheeler algorithm (BWA) (v0.7.17 (r1188)) *mem* (Li, 2013) method with default parameters. BWA-generated alignment files were sorted and deduplicated by using the Picard package (v2.1.1). Then, variants were called for each sample independently using the Sentieon (Freed et al., 2017) DNAseq Haplotyper and generated the genomic Variant Call Format (gVCF) format files. Joint genotyping was performed on 45 gVCF files using the Sentieon DNAseq GVCFtyper. This step creates a common VCF file having the information from all the 45 individuals with 48,359,621 single-nucleotide polymorphisms (SNPs) and 8,243,306 insertions/deletions (indels). We firstly removed indels and then performed hard filtering with “QD < 2.0 || FS greater than 60.0 || MQ < 40.0 || MQRankSum < −12.5 || ReadPosRankSum < −8.0 --filter-name snp_filter” (DePristo et al., 2011). To facilitate downstream analysis, we also filtered multiallelic variants.

### Genome-Wide Genetic Diversity and Population History

The genome-wide heterozygosity (*H*) used for assessing the genetic diversity was calculated based on autosomal SNPs using VCFtools (v4.1) (Danecek et al., 2011). We first inferred the demographic history of *P. larvata* using the pairwise sequentially Markovian coalescent (PSMC) (Li and Durbin, 2011) method. The analysis was carried out with 64 atom time intervals under the pattern of “4 + 25 × 2 + 4 + 6”. The estimated theta values were then transformed to effective population sizes and plotted with a generation time (g) of 2 years and the mutation rate (μ) of 2.4 × 10^–9^ substitution per site per generation (Yu et al., 2021). For each individual, 100 bootstrap replicates were performed to evaluate the robustness of the estimation. To resolve more recent demographic histories clearly and robustly, we performed SMC++ (Terhorst et al., 2017) analysis based on the population variations from the 45 individuals. We used the same g and μ in the SMC++ analysis with the PSMC analysis. We did not perform the MSMC2 (Schiffels and Durbin, 2014) analysis here because we cannot control the phasing errors in the population we used in this study.

### Gene Expression Analysis

Gene expression was estimated through the Trinity pipeline (v2.11.0), with transcript quantification by RNA sequencing by expectation maximization (RSEM) (Li and Dewey, 2011; Haas et al., 2013). We first obtained high-quality reads by removing adaptor sequences and low-quality reads by Trimmomatic (v0.33.0) (Bolger et al., 2014). Then, clean transcript data was mapped to the *P. larvata* genome to determine the gene locus and estimate abundance using align_and_estimate_abundance.pl with parameter of “--est_method RSEM--aln_method bowtie2” (Langmead and Salzberg, 2012). Transcript abundance was normalized using the transcripts per million (TPM) method. Genes expression heatmap was generated using R package “pheatmap” (https://CRAN.R-project.org/package=pheatmap), with row (i.e., TPM of viral receptor genes) scaling by “scale = row” parameter.

## Results

### Chromosome Scale Genome Assembly

The genome size of the *P. larvata* was estimated to be 2.46 Gb, with a heterozygosity rate of 0.635% (Supplementary Figure S1), by calculating the frequency of 17-mer using 82.08 Gb short reads generated by DNBSEQ-T1 sequencer. We finally generated 483.51 Gb stLFR sequencing data from two long fragment genomic DNA libraries and 105.94 Gb Hi-C generated data from one genomic DNA library for the chromosome-scale genome assembly (Table 1).

**TABLE 1:** Global statistics of sequencing data, genome assembly, and annotation of *P. larvata*.

Item	Category	Number
Sequencing data	stLFR (Gb)	483.5
	Genome survey (Gb)	82.08
	Hi-C (Gb)	105.94
	Re-sequencing (45 individuals) (Gb)	2,593.62
	RNA-seq (16 organs) (Gb)	1,022.6
Assembly (Hi-C)	Estimated genome size (Gb)	2.46
	Assembled genome size (Gb)	2.42
	Karyotype	2n = 44
	Assembly chromosome number	22
	Contig N50 (kb)	77.18
	Scaffold N50 (Mb)	105.6
	Longest scaffold (Mb)	190.64
Annotation	GC content (%)	42.1
	Repeat sequences (%)	32.64
	Number of protein-coding genes	18,340
	Number of functionally annotated genes	18,333
	Average gene length (kp)	40.67
	Average exon length (bp)	182.84
	Average intron length (kb)	5.10
	Average exon per gene	8.67

The primary genome was first assembled by using stLFR sequencing data, with the contig N50 and scaffold N50 of 80.64 kb and 8.76 Mb, respectively. We then concatenated primary scaffolds to chromosome-scale assembly by using Hi-C sequencing data. The final genome size after redundancy removal was 2.42 Gb, accounting for 98.37% of the estimated genome. The genome of the *P. larvata* contains 22 pairs of chromosomes (2n = 44), including 21 pairs of autosomes and 1 pair of allosome (Tanomtong et al., 2011). Here we assigned 2.18 Gb of the genome region to 22 chromosomes (Supplementary Figure S2), with the final scaffold N50 of 105.60 Mb. BUSCO (Simão et al., 2015) analysis showed that 92.1% of 4,104 Mammalia BUSCO genes were identified in our genome, with 0.6% duplicated and 91.5% single copy, and the remaining 3.5% and 4.4% were identified missing and fragmented (Supplementary Table S2). Besides, the guanine and cytosine (GC) content of this genome is 42.1%, which was very similar to 42.5% of its closely related species, *Paradoxurus hermaphroditus* (GCA_004024585.1). In addition, 94.53%, 98.94%, 99.33%, and 99.20% of transcript data, WGS short reads, stLFR sequencing data, and Hi-C generated reads can be mapped onto our final assembly, respectively. All the above assessments showed a complete genome with high quality and contiguity.

**FIGURE 1:**
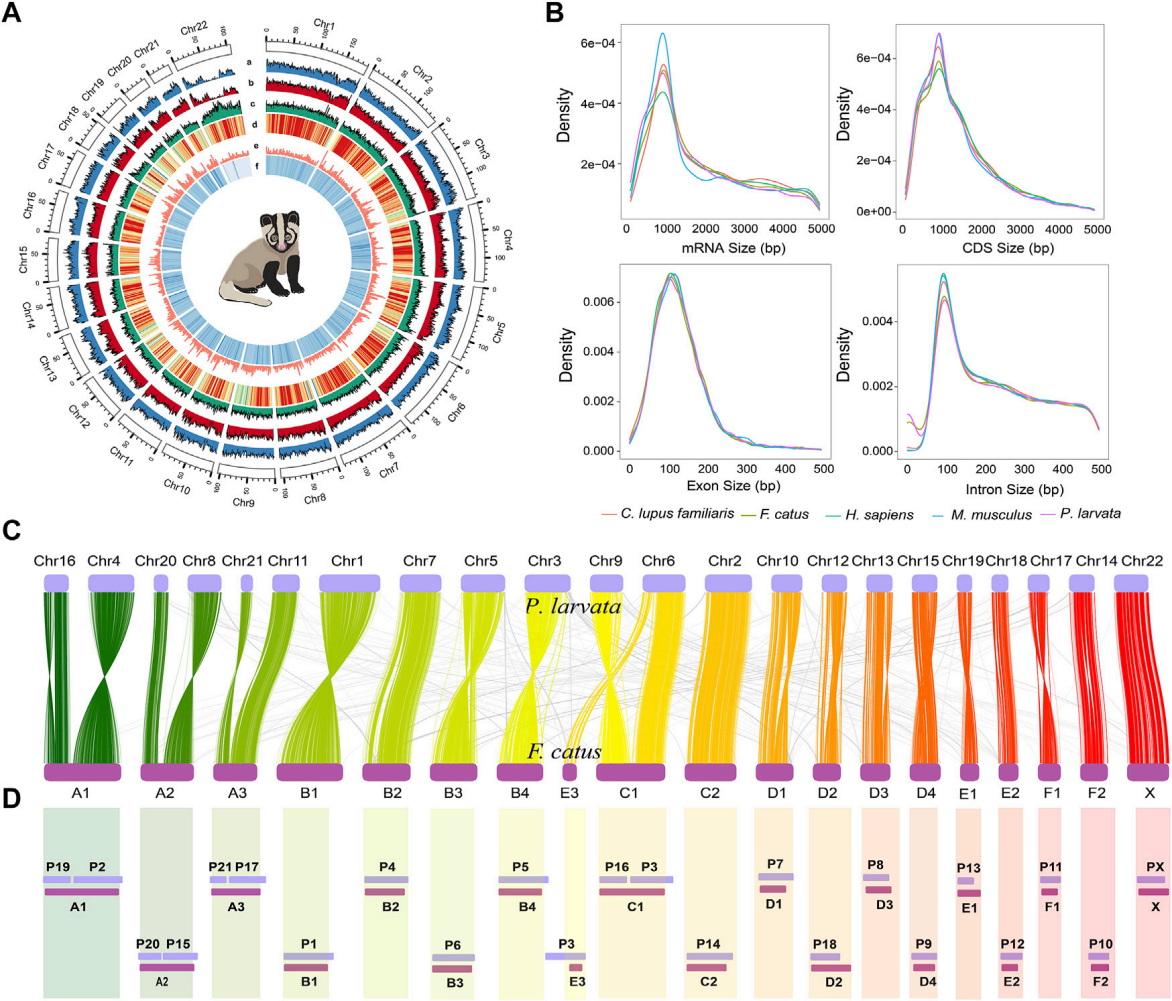
Overview of *P. larvata* genome assembly and chromosome-level synteny analysis in this study. **(A)** Genomic features and variation landscape of our assembled genome (500 kb window). a, population-scale π-values across 22 chromosomes; b, SNP counts per 500 kb window; c, repeat density; d, GC content; e, gene density; f, read depth mapped to the genome. **(B)** Comparisons of mRNA length, coding sequence (CDS) length, exon length, and intron length among the five species. The x-axis represents the length, and the y-axis represents the density. **(C)** Chromosome-level synteny analysis between *P. larvata* and *F. catus*, which was visualized using RectChr v1.27 (https://github.com/BGI-shenzhen/RectChr). **(D)** Schematic diagram of a comparative chromosome map of *P. larvata* (P chromosomes) and *F. catus* (A–F chromosomes) from previously published karyotypic study (Perelman et al., 2005). Lower bands represent the cat's chromosomes, and the upper bands represent the masked palm civet's chromosomes. All fission and fusion events found in **(C)** can be correspondently found in **(D)**. We assume that the chromosome relationships between the cat and masked palm civet was accurate in the karyotypic analysis, then the highly consistent result between karyotypic and the syntenic analysis indicates the high quality of our assembled chromosome-scale genome.

Further, we performed the inter-species synteny analysis between the genome of *P. larvata* and that of the cat (*F. catus*, GCA_000181335.4). In general, we found high collinearity between the two genomes with clear one-to-one blocks found in Figure 1C. But the fusion and fission events were still observed in this analysis. The chromosome Chr6 of the *P. larvata* was identified to be the fusion of E3 and C1 of *F. catus*. A1, A2, A3, and C1 were all split into two chromosomes in the *P. larvata* genome*.* All these fission and fusion events were surprisingly consistent with the previous karyotypic study (Perelman et al., 2005), indicating the high quality of our assembled chromosome-scale genome.

### Genome Annotation

Usually, the repeat elements are widely distributed across the whole genome in eukaryotic genomes and play important roles in evolution. We identified 782.98 Mb repetitive elements in our assembled *P. larvata* genome, representing 32.64% of the total genome size. The most abundant repeat category was LINEs (85.22%), followed by LTRs (20.27%), DNA elements (5.19%), and SINEs (1.71%) (Supplementary Table S3–S5, Supplementary Figure S3). We masked all these repeat sequences for genome annotation.

By combining evidence from *ab initio* prediction, transcript mapping, and homology-based protein mapping, we predicted a total of 18,340 high confident protein coding genes, which was generally consistent with gene numbers annotated in other carnivores (Li et al., 2010; Cho et al., 2013; Dobrynin et al., 2015; Armstrong et al., 2020). Gene features were also highly consistent with other mammals, with an average gene length, intron length, and exon length of 40.67 kb, 5.10 kb, and 182.84 bp (8.67 exons per gene), respectively (Figure 1B and Supplementary Table S6). Among these genes, 15,535 (∼84.7%) were supported by transcript data. Also, 92.7% and 2.5% of the complete BUSCOs and fragmented BUSCOs were identified in the BUSCO analysis, respectively, showing the high completeness of our predicted gene set. Finally, 18,333 (99.96%) protein coding genes were functionally annotated in at least one of the five databases we used in the method part (Supplementary Table S7, Supplementary Figure S4). In addition, 1,097 miRNA, 714 rRNA, 45,835 tRNA, and 1942 snRNA were predicted in the whole *P. larvata* genome (Supplementary Table S8).

### Identification of Sex-Linked Regions

**FIGURE 2:**
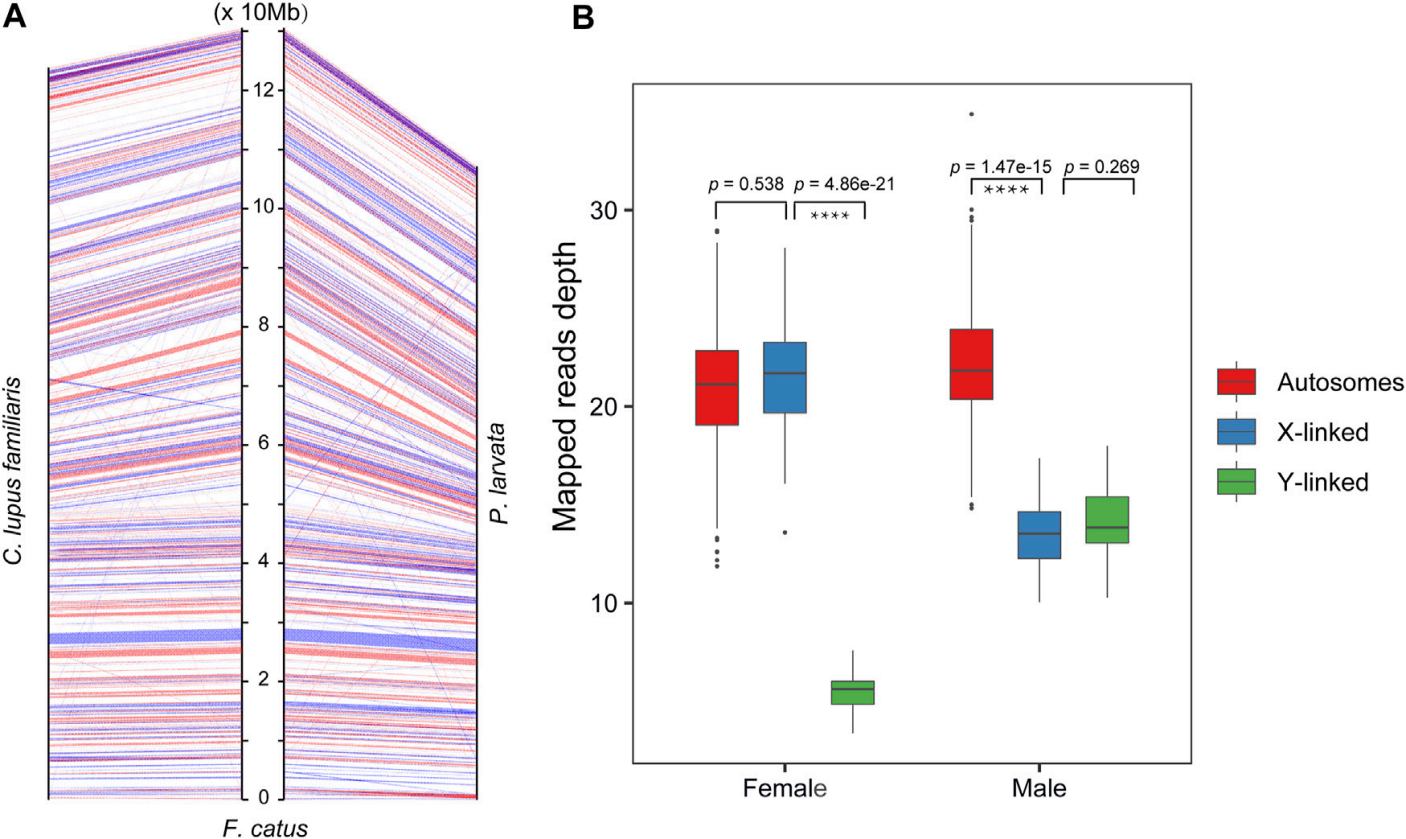
The identification of sex-linked regions in this study. **(A)** Anchoring genes on the X chromosome of the *F. catus* to the *P. larvata* and *C. lupus familiaris*. **(B)** The read depth mapped to autosomes, X-linked region, and Y-linked region of the 45 re-sequenced *P. larvata* individuals.

To identify the sex-linked regions in the genome of *P. larvata*, we first mapped our assembled whole genome to the female reference assembly (GCA_000181335.4) and the Y chromosome sequence (KP081775) of the *F. catus*. This comparison showed that the X chromosome of the *F. catus* was highly similar to the Chr22 of *P. larvata* (Figure 1C). Considering the high collinearity of the gene set on the X chromosome between the cat and dog, we further mapped the 796 genes on the X chromosome of the cat to the *P. larvata* genome. In total, 795 (99.87%) genes were found in our assembled genome, among which 753 (94.6%) genes could be detected on the Chr22, supporting that the Chr22 was originated from the X chromosome (Figure 2A, Supplementary Table S9).

For the identification of Y-linked regions, however, it is more challenging because of the poor collinearity when compared with the cat genome. We also found poor collinearity of the Y chromosomes between the *F. catus* and *Canis lupus familiaris* (KP081776), suggesting that the homology of Y chromosomes among these species is low, which may largely be due to the high proportion of repeat sequences and lots of rearrangements in the Y chromosome (Ventura et al., 2012). The sex determining region of the Y chromosome (*SRY*) is a transcription factor that is responsible for testis determination, which is believed to be evolutionary conserved among mammalian Y chromosomes, especially for the high mobility group (HMG) box region (Ely et al., 2010). We then used the SRY gene as the marker to identify Y-linked regions and found that this gene was located on the Scaf457. We considered that the Scaf457 was from the Y chromosome. Further detection found five other Y-linked genes were also located on this scaffold, including *KDM5D*, *USP9Y*, *HSFY*, *DDX3Y*, and *UBE1Y* genes. We then identified that the Scaf457 was a Y-linked region.

To further testify the identification of the X and Y chromosomes, we checked the read depths that mapped to the autosomes and the two identified sex-linked genome regions by mapping the whole genome sequencing reads from the other 45 individuals (18 male and 27 female individuals) to our assembled genome (Supplementary Table S10). As we expected, the sequencing depth of the Chr22 and Scaf457 in the male individuals were nearly half of the autosomes (Figure 2B, Supplementary Table S10). For the female individuals, however, the depths of the Chr22 were nearly the same as the autosomes (Figure 2B, Supplementary Table S10). These results further supported that our identification of the sex-linked regions was accurate. In brief, we identified 107.13 Mb X-linked regions and 1.34 Mb Y-linked regions. This was the first time we identified sex-linked genome regions in the *P. larvata*, which will be a valuable data source for future related studies.

### Investigation of the Possible Genetic Basis for Its Omnivorous Diet

**FIGURE 3:**
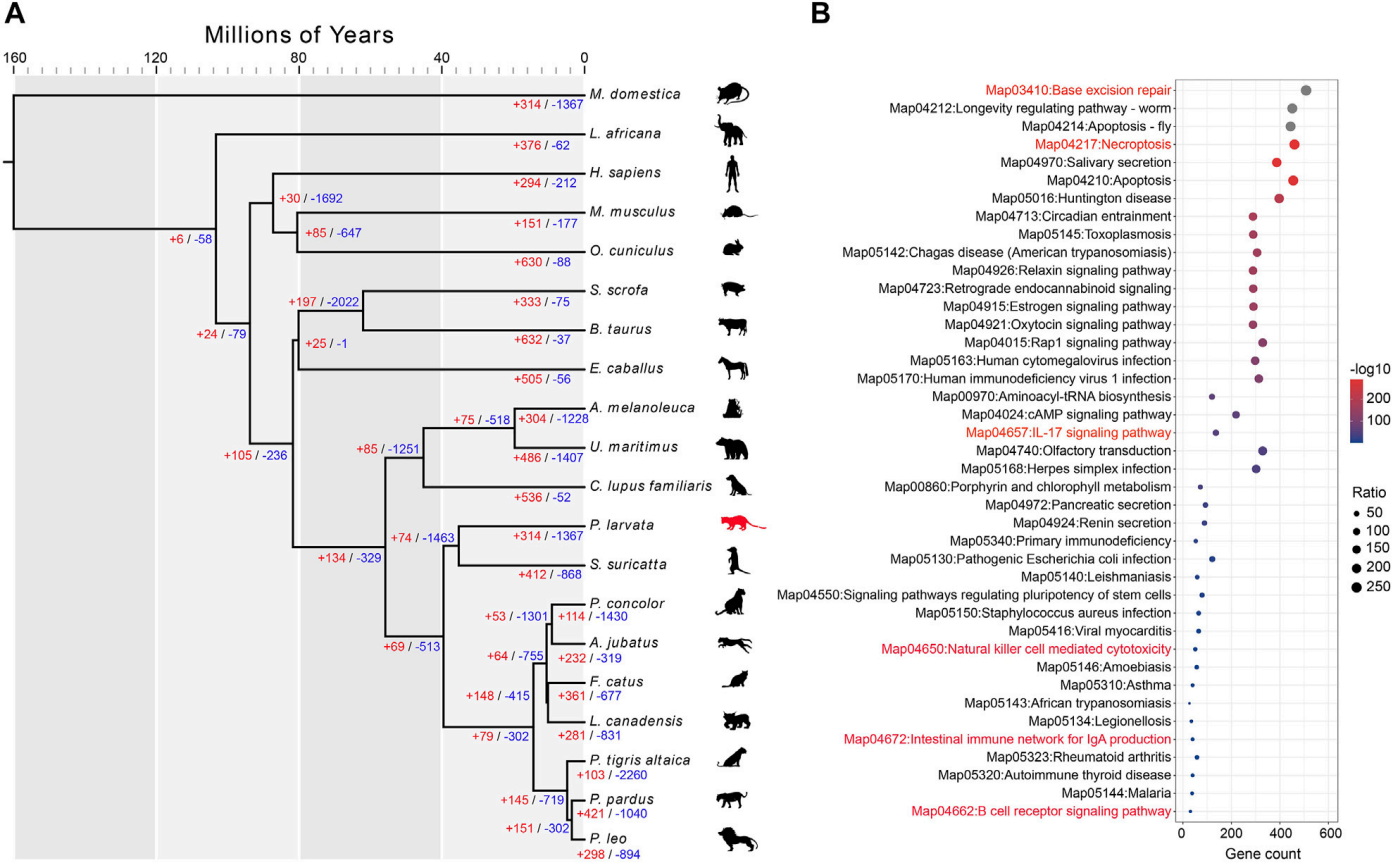
Comparative genomic analysis and enrichment analysis of expanded gene families. **(A)** Divergence time estimated among 20 species. This tree topology was generated by MCMCtree with CDS sequences. The red and blue numbers indicate the expanded and contracted gene families for each node. Illustrations were created by adapting SMART (https://smart.servier.com) and Vecteezy (vecteezy.com) templates. **(B)** The significantly enriched KEGG pathways of 314 expanded gene families in the *P. larvata* genome compared with 19 other species.

The *P. larvata* belongs to the order Carnivora, but they are omnivores with a broad spectrum of prey items (Zhou et al., 2008; Iwama et al., 2017). To explore the genomic adaptations of their omnivorous diet, we performed extensive comparative genomics analysis along with 19 other species, including 9 carnivores, 5 omnivores, and 5 herbivores (Supplementary Table S1). We first detected the expanded and contracted gene families in the genome of the *P. larvata* compared to the common ancestor with the *Suricata suricatta*. The gene family expansion and contraction analysis showed that 314 gene families, including 2,209 genes, were detected expanded and 1,367 gene families were detected contracted (Figure 3A). The *S. suricatta* is a typical meat-eating carnivore, and we expected to find expanded gene families in the *P. larvata* that might be related to detoxification and polyphagia considering its plant diet. We then focused on the following gene families, including cytochrome P450 (CYP450) (Johnson et al., 2018), carboxylesterase (CES) (Holmes et al., 2010), glutathione S-transferase (GST) (Hayes and Pulford, 1995), ATP-binding cassette (ABC) (Koenig et al., 2015), protein tyrosine phosphatase (UGT), aldehyde oxidase (AOX) (Chang et al., 2010), taste receptor type 2 (TAS2R) (Jiang et al., 2012), and amylase (AMY) (Kim et al., 2016) gene families. Unexpectedly, none of the above mentioned gene families were part of the 314 expanded gene families (Supplementary Table S11, Supplementary Table S15). We further found 28 expanded gene families compared with the 7 meat-eating carnivores (Supplementary Table S13, Supplementary Table S17), but we still could not find any expanded gene families mentioned above, as well as other gene families related to detoxification and polyphagia. However, GO and KEGG enrichment analysis found that the olfactory receptor activity (GO:0004984, *p* = 2.28E-182), salivary secretion (map04970, *p* = 4.08E-289), and olfactory transduction (map04740, *p* = 7.83E-49) were significantly enriched in the genome of the *P. larvata* (Supplementary Table S11–S18), which appeared to be related with feeding habits.

**FIGURE 4:**
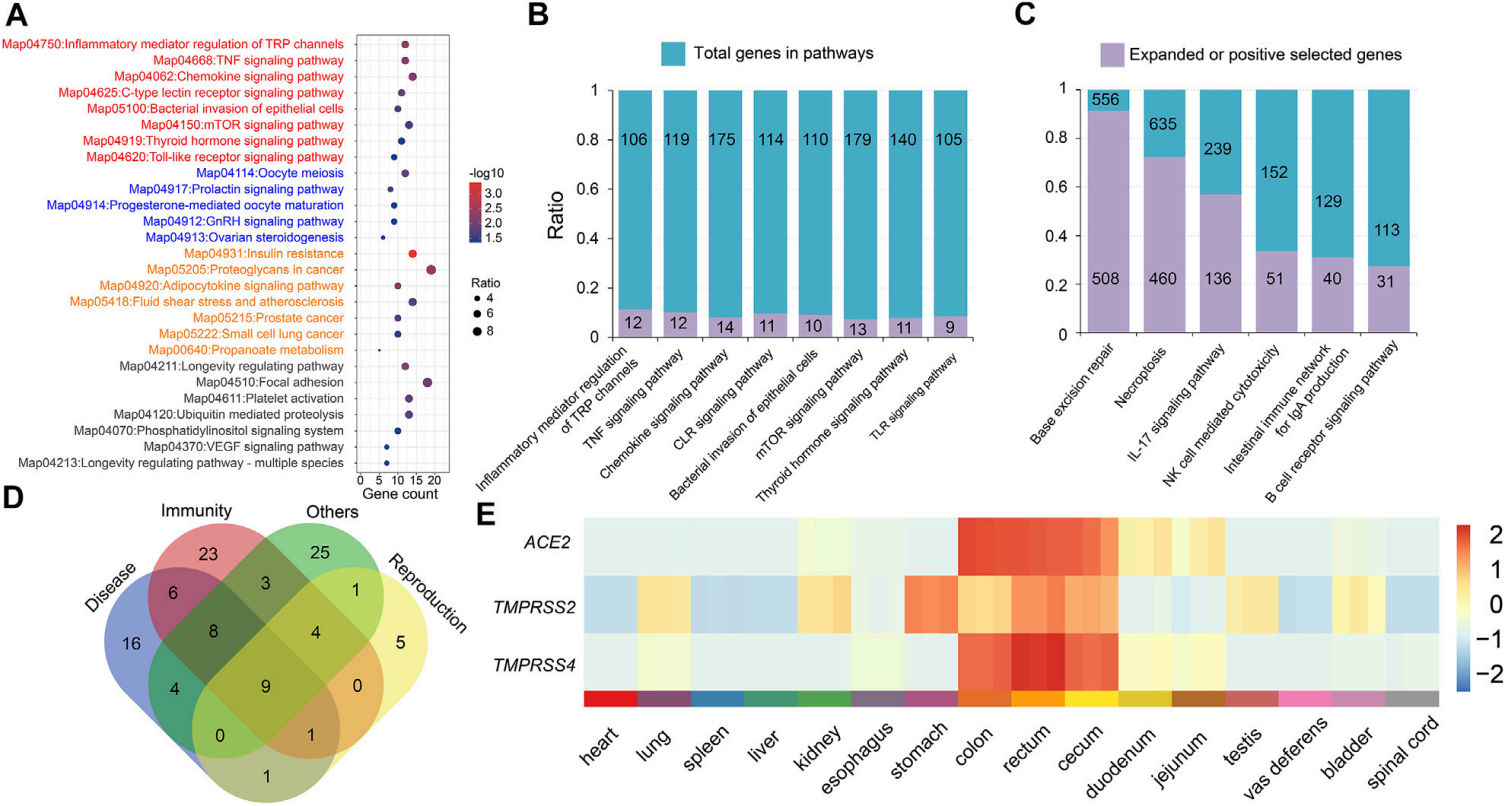
Enriched KEGG pathways related to the immune system and the RNA expression profile of *ACE2* and *TMPRSS2/TMPRSS4* genes. **(A)** Enriched KEGG pathways of the 622 PSGs of the *P. larvata* compared with 19 other species. Red, pathways related to immunity; blue, pathways related to reproduction; orange, pathways related to disease. **(B)** The number of PSGs in the eight significantly enriched KEGG pathways related to the immunity. **(C)** The number of genes in the expanded gene families in the six enriched KEGG pathways related to the immunity. **(D)** The gene number of PSGs in significantly enriched KEGG pathways related to immunity, reproduction, and disease. Some genes were shared in different pathways. **(E)** The expression heatmap of *ACE2*, *TMPRSS2*, and *TMPRSS4* genes in 16 organs of the masked palm civet. Z-scores were calculated from TPM values for each of the three genes. Gene expression is colored from low (blue) to high (red).

We continue to detect genes evolving under positive selection for its general diet with 19 other species. A total of 622 genes were detected under positive selection in the *P. larvata.* We then performed the GO and KEGG enrichment analysis on these PSGs. Again, we did not find direct and obvious evidence on its general diet with GO enriched in protein binding (GO:0005515, *p* = 0.002693691), catalytic activity (GO:0003824, *p* = 0.01071653), golgi transport complex (GO:0017119, 0.011630804), binding (GO:0005488, *p* = 0.024289689), helicase activity (GO:0004386, *p* = 0.03616881), transferase activity (GO:0016740, *p* = 0.042327718), and KEGG mainly enriched in immunity, diseases, and reproduction (Figure 4A). Actually, we still detected that the *ABCD3* and *CYP450 2U1* genes were under positive selection. The *ABCD3* gene was a member of the ABC gene family, and the *CYP450 2U1* gene was from the CYP450 gene family. We also performed the positive selection analysis with the meat-eating Felidae species, and we obtained similar result (Supplementary Table S19–S22).

We speculated that the *P. larvata* can taste sweet due to its fruit diet. *TAS1R2/TAS1R3* mediated sweet taste in mammals; we checked these two genes in the *P. larvata* genome and expected to find both intact and functional genes. However, we found that the *TAS1R2* gene in the *P. larvata* genome was incomplete, and the transcriptomic data of this gene was also fragmented, so that we inferred that this was a pseudogene in the *P. larvata* genome, just like the pure meat-eating Felidae animals (Supplementary Figure S5).

We finally constructed the phylogenetic tree by using the above mentioned 9 gene families with all the above mentioned 19 species (Supplementary Table S1). Genes in each gene family of the *P. larvata* seemed to be randomly distributed in phylogenetic trees without expansion, and species-specific structures were not found (Supplementary Figure S6–S14).

### Exploring Genomic Cues Regarding the Immunity of the *P. larvata*

Considering that the *P. larvata* is the vector of many pathogens (Shi and Hu, 2008; Lee et al., 2011; Sato et al., 2013; Hou et al., 2016; Wicker et al., 2017; Yu et al., 2020) but with fewer reported symptoms (Wicker et al., 2017), we expected that the *P. larvata* may have a strong immune system to protect themselves from pathogens. We then explored the genomic signatures for its adaptation against different pathogens.

Functional enrichment analysis on expanded gene families showed that 183 GO items were significantly enriched (*p* < 0.05) in the *P. larvata* genome (Supplementary Table S11). The most significantly enriched GO items included the ribosome (GO:0005840, *p* = 1.19E-226), structural constituent of ribosome (GO:0003735, *p* = 5.57E-226), ribonucleoprotein complex (GO:1990904, *p* = 3.74E-211), structural molecule activity (GO:0005198, *p* = 5.00E-193), *etc.* We did not find enriched GO items that were directly related to the immune system. In the 57 significant enriched KEGG pathways (Figure 3B, Figure 4C), however, we found several significantly enriched pathways that were directly related to the immune system, including IL-17 signaling pathway (map04657, *p* = 2.61E-54), natural killer (NK) cell mediated cytotoxicity (map04650, *p* = 3.92E-06), intestinal immune network for IgA production (map04672, *p* = 0.000447), and B cell receptor signaling pathway (map04662, *p* = 0.020766). Besides, we have found two other enriched pathways that may be related to immune defense, including base excision repair (BER) (map03410, *p* = 0, FDR-adjusted) and necroptosis (map04217, *p* = 1.09E-291) (Figure 4B).

Functional enrichment analysis on the 622 PSGs showed that 27 KEGG pathways were significantly enriched (Figure 4A). It is noteworthy that there were 107 genes in these 27 pathways; 54 genes (50.47%) in eight pathways (29.63%) were related to immunity (Figures 4B,D; Supplementary Table S21). The most related pathways were tumor necrosis factor (TNF) signaling pathway (map04668, *p* = 0.008023), chemokine signaling pathway (map04062, *p* = 0.008023), C-type lectin receptor (CLR) signaling pathway (map04625, *p* = 0.014906), and toll-like receptor (TLR) signaling pathway (map04620, *p* = 0.043078).

### Prediction of the Most Possible Viral Entry Routes of the SARS-CoV-2

It is well known that *P. larvata* is a famous intermediate host of the SARS-CoV virus (Guan et al., 2003). Prediction of the possible viral entry routes of the SARS-CoV-like viruses can greatly help to control the transmission between humans and animals. ACE2 and TMPRSS2/TMPRSS4 were reported to help SARS-CoV-2 enter into cells by binding and priming of this virus (Hoffmann et al., 2020; Zang et al., 2020). In this study, we systematically screened the gene expression of *ACE2*, *TMPRSS2*, and *TMPRSS4* genes in 16 organs of the *P. larvata* (Supplementary Table S24). We found that these three genes were expressed in all these 16 organs with different expression levels. In general, the co-expression of the *ACE2* and *TMPRSS2/TMPRSS4* was found the most obvious in the rectum, colon, and cecum (Figure 4E). Although *P. larvata* has been predicted as low susceptible to the SARS-CoV-2 (Damas et al., 2020), we still warned that the SARS-CoV-2 was more likely to infect the *P. larvata* through the rectum, colon, and cecum, providing valuable information for the management of this species to prevent SARS-CoV-2 from being transmitted to humans.

### Population Decline With High Genomic Diversity

**FIGURE 5:**
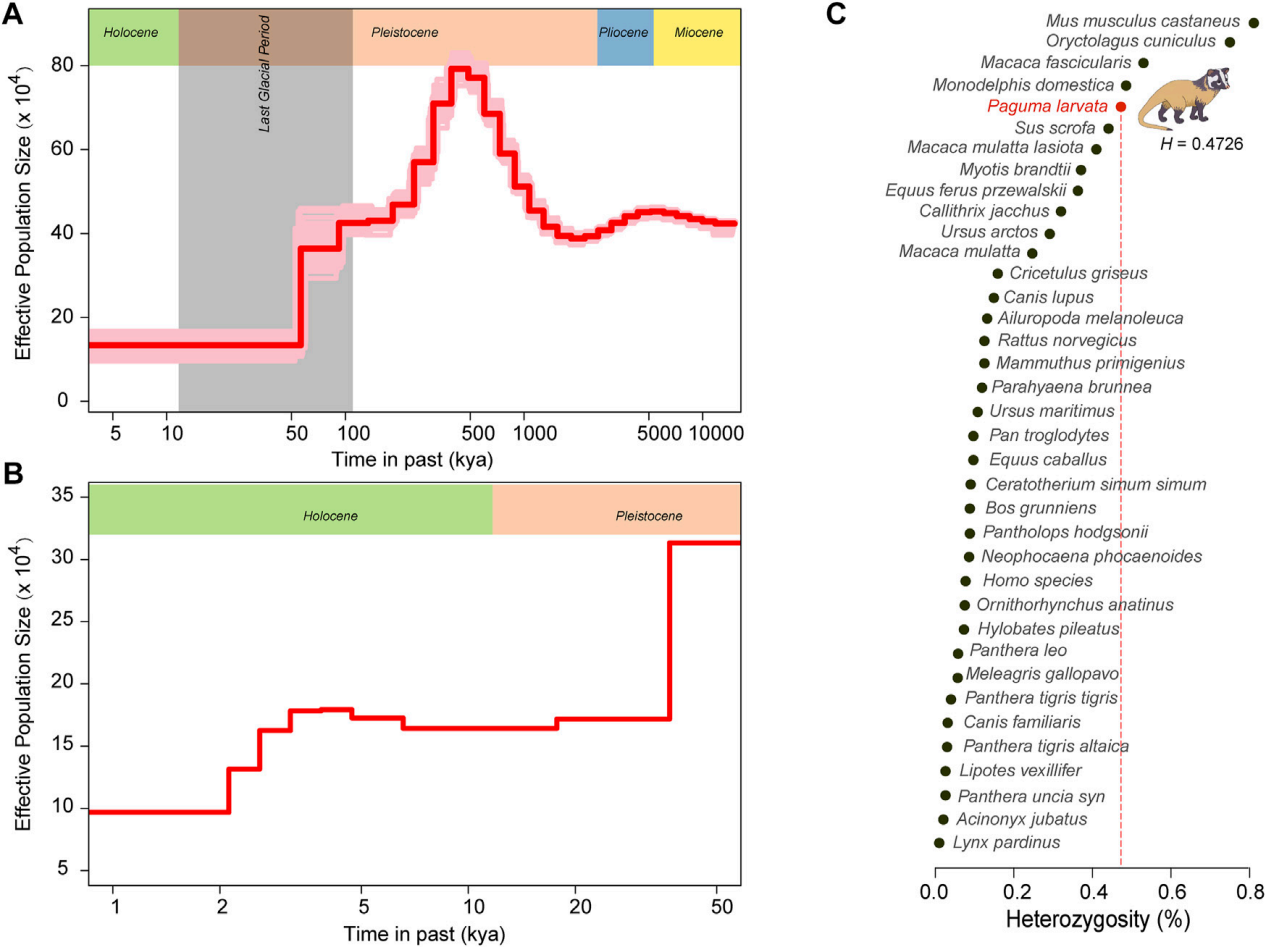
Genomic heterozygosity and population history of the *P. larvata*. **(A)** Demographic history of *P. larvata* estimated by PSMC. **(B)** Demographic history of *P. larvata* estimated by SMC++. **(C)** The genome-wide heterozygosity in *P. larvata* and 36 other published species. The generation interval and mutation rate we used here were 2 years and 2.4 × 10^–9^.

To explore the genetic diversity of this species, we calculated the genome-wide heterozygosity (*H*) of the *P. larvata* and compared it with the other 36 species. The *H* of the *P. larvata* was estimated to be 0.4726, which is several times larger than that of many endangered species, such as the giant panda (*H* = 0.132), tiger (*H*_*Bengal tiger*_ = 0.04; *H*_*Siberian tiger*_ = 0.03), African lion (*H* = 0.058), snow leopard (*H* = 0.023), brown bear (*H* = 0.32), polar bear (*H* = 0.108), and southern white rhinoceros (*H* = 0.09) (Figure 5C, Supplementary Table S25). This value is even larger than that of Han Chinese (*H* = 0.077), wild boar (*H* = 0.441), and gray wolf (*H* = 0.149), which are not endangered at all.

To further investigate the population dynamics over its evolutionary history, we performed the PSMC and SMC++ analyses to systematically describe the changes in its effective population size over time. The result from PSMC showed that this species experienced two population expansions and two population contractions. The first expansion occurred at the remote Miocene, and the second population expansion occurred between 2.5 and 0.5 million years ago (Mya). The two population decline events were detected from 5.5 to 2.5 Mya and from 0.5 Mya to the present day, respectively (Figure 5A). The PSMC cannot accurately infer the recent population history (Li and Durbin, 2011). Therefore, we focused on the result from SMC++ to further explore its recent population history (within 10,000 years). From Figure 5B, we found a more recent decrease of the effective population size at ∼4 thousand years ago (kya). Although the effective population size is continuously declining since 500 kya, the real effective population size was still large with nearly 100,000 individuals at the lowest point.

## Discussion

### Chromosome-Scale Genome Assembly Provides Valuable Genetic Resource

The *P. larvata* is a small mammal with several distinct characteristics related to its evolution and survival. For example, taxonomically it is a carnivore species but with an omnivorous diet, and it is a vector of pathogens but with fewer symptoms reported (Wicker et al., 2017). Particularly, the public was extremely concerned by the *P. larvata* as the SARS-CoV was identified from this species (Guan et al., 2003). With the rapid development of the genome sequencing technology and the plummeting cost of sequencing, a large number of mammal genomes have been sequenced for exploring the possible genetic basis for biological questions. However, the genome of the *P. larvata* is still not characterized yet. Particularly, the *TMPRSS2* gene in the *P. larvata* genome was reported lacking, which is possibly due to the genome incompleteness (Huang et al., 2021). Here, we assembled a chromosome-scale genome of the *P. larvata*, which is the first genome of this species. The total genome size is 2.42 Gb, which is comparable with other carnivores, including the giant panda (2.25 Gb) (Li et al., 2010), Amur tiger (2.4 Gb) (Cho et al., 2013), lion (2.4 Gb) (Armstrong et al., 2020), cheetah (2.36 Gb) (Dobrynin et al., 2015), and cat (2.52 Gb) (Buckley et al., 2020). Particularly, we annotated the *TMPRSS2* gene in the gene set, which was reported lacking in the *P. larvata* genome (Huang et al., 2021), indicating the advantage and necessity of the this assembly. Besides, we identified 107.13 and 1.34 Mb X- and Y-linked regions in this genome by combining several methods followed by validation using genome resequencing data from 45 individuals. We believe that this genome will provide a valuable resource to promote the research on *P. larvata.*

### No Obvious Genomic Basis Was Found for Its Dietary Shift

As mentioned above, the *P. larvata* is a small carnivore but with an extremely broad spectrum of prey items, including both animals and plants, and even takes plant cortexes and leaves as food (Zhou et al., 2008; Iwama et al., 2017). To explore the possible genetic basis for their omnivorous diet, we first focused on gene families related to detoxification, because there are many toxic secondary metabolites in plants (Xia et al., 2021). Besides, the poisonous substances are typically bitter in taste, such as plant alkaloids (Li and Zhang, 2014); we then examined the bitterness taste receptor gene family (TAS2R). Considering that the omnivores should have the stronger ability to use starch, we also took the AMY gene families into consideration. Basically, we focused on nine gene families to explore the possible genetic basis of its omnivorous diet, including CYP450, CES, GST, ABC, UGT, AOX, TAS2R, TAS1R, and AMY gene families. The expansion of CYP450 and taste receptor gene families was reported to help koalas cope with poisonous eucalypt foliage (Johnson et al., 2018). However, none of these gene families were found to be significantly expanded in the *P. larvata* genome. However, we detected the olfactory receptor activity, salivary secretion, and olfactory transduction were significantly enriched in the genome of the *P. larvata* (Supplementary Table S11, Supplementary Table S15), which seem to be related to the more general diets when compared with meat-eating carnivores. Further examination showed that these three gene families were also detected being expanded when compared with omnivores and herbivores (Supplementary Table S12, S14, S16, S18), suggesting that these expanded gene families might be related to other functions but not the omnivorous diet of the *P. larvata.*

Based on the enrichment analysis of the 622 PSGs, we were unable to determine whether these gene families were significantly enriched. But the *CYP450 2U1* and *ABCD3* genes were detected under positive selection. The ABC and CYP450 gene families are all closely related with detoxification, which may be important for plant-eating animals to protect themselves from toxicants in the plant (Koenig et al., 2015; Johnson et al., 2018). However, these two genes were not directly related to detoxification. The *CYP450 2U1* gene and ABCD subfamily were both conserved among species, and the *CYP450 2U1* was mainly involved in the fatty acids, whereas the ABCD subfamily was mainly involved in the peroxisomal pathways (Devos et al., 2010; Wu et al., 2019). We still did not find enriched GO categories or KEGG pathways that are directly related to its omnivorous diet by positive selection analysis with pure meat-eating Felidae animals. The pseudogenized *TAS1R2* gene and not expanded TAS2R gene family both showed that the taste receptor genes may not be the direct genetic causes for its omnivorous diet. Considering the diet shift might be a transition state in the *P. larvata*, we constructed phylogenetic trees by using the above mentioned 9 gene families with 19 other species, including omnivores, herbivores, and carnivores. No abnormal phylogenetic relationships were observed. Taken together, we failed to find a strong genetic basis for the omnivorous diet of this species. We speculated that epigenetics or metagenomics might contribute more to its omnivorous diet than genomics. Another possibility is that the *P. larvata'*s diet is general, but they eat much less plant cortexes and leaves than other food, or they have evolved the ability to only feed on plants without poisonous substances through their evolutionary history.

### Reinforced Immune System in the *P. larvata*

The *P. larvata* has been reported as the vector for many pathogens, including the SARS coronavirus (Shi and Hu, 2008; Lee et al., 2011; Sato et al., 2013; Hou et al., 2016; Wicker et al., 2017; Yu et al., 2020). The immune function is of vital importance for animals for defense against foreign pathogens (Chaplin, 2010). In this study, we expected that the *P. larvata* has a strong immune system to protect themselves from being infected by pathogens. In the expanded gene families, several biological pathways were detected to be significantly enriched, including interleukin, NK cell, IgA production, and B cell receptor related pathways, which were all directly related to the immune ability to defend against foreign pathogens. Besides, we also found the BER and necroptosis pathways were significantly enriched in the *P. larvata* genome with large amount of expanded gene families in these two pathways (Figure 3B, Figure 4C). Further investigation showed that the BER pathway plays an important role in not only preventing cancer but also the immune tolerance. Defects in this pathway were found to be associated with autoimmune diseases (Stratigopoulou et al., 2020). Innate immunity could be activated by necroptosis through inducing cell death or releasing signals to provoke the immune system to clear pathogens (Cho, 2020). These enriched biological pathways in the *P. larvata* genome may be related to the immune defense to protect it from pathogen attacks. The positive selection analysis further led to the identification of a large proportion of significantly enriched pathways and genes that were immune system related. The most relevant pathways were related to the TNF, chemokine, CLR, and TLR. TNF is a key regulator of immune responses involved in mediating cell death and cell survival to influence the function of the immune system. Severe inflammatory diseases will potentially be induced by disturbing the signaling pathway (Webster and Vucic, 2020). The chemokines are also critical inflammatory response mediators which can regulate cell recruitment in both the adaptive and innate immune systems to defend against pathogens (Hembruff and Cheng, 2009). CLRs play crucial roles in tailoring immune responses to various pathogens like fungi, bacteria, parasites, and viruses (Hoving et al., 2014). Dysregulation of CLRs will result in inflammatory diseases (Drouin et al., 2020). Like the above three pathways, the TLR signaling pathway is also an important member of the immune system. TLRs are widely expressed in immune cells to help recognize pathogens and detect early infections, and they can bridge the adaptive and innate immunities (Maglione et al., 2015). Taken together, we speculated that these expanded gene families and positively selected genes were, at least to some extent, related to the immune defense of the *P. larvata* as a pathogen vector. However, the exact function and relationship between these genes and gene families still needed to be further investigated and confirmed by other designs like expression pattern and functional validations.

### Declining Population With High Genomic Diversity

Although *P. larvata* is currently listed as the least concerned species in the International Union for Conservation of Nature (IUCN) Red List of Threatened Species, its population is continuously declining, which is likely due to the loss of forest in recent decades (Jennifer and Todd, 2014). Patou et al. investigated the genetic diversity of the *P. larvata* from China, the Sundaic region, and the Indochinese region by using mitochondrial DNA fragments, indicating a low genetic diversity (Patou et al., 2009). In this study, however, the genome-wide *H* of the *P. larvata* was quite high when compared with both endangered and unendangered animals (Figure 5C), indicating a relatively healthy genetic status of this species. In future, more individuals from its natural distribution areas should be collected to evaluate the population-scale genome-wide genetic diversity to fully compare our results to Patou's study. Interestingly, the effective population size of this species has continuously declined over the past ∼500 kya, and an obvious decline occurred at around ∼4 to 5 kya. Considering the early human activity was much later than 500 kya, we infer that the decline of the effective population of the *P. larvata* might have resulted from both climate and human activities. It is noteworthy that the high *H* and the long-term population decline is controversial. But further examination showed that the real effective population size was still large with nearly 100,000 individuals in the lowest point, which is much larger than that of many endangered animals (Cho et al., 2013; Zhao et al., 2013; Paijmans et al., 2021). Besides, we found that a large amount of enriched KEGG pathways on the expanded gene families and PSGs were related to both reproduction and diseases (Figure 4A, Figure 4D), indicating that there may exist mechanisms to keep a fast population turnover in this species by balancing birth and death. These factors we inferred might be important to sustain a high genetic diversity.

## Data Availability

The data that support the findings in this study have been deposited into CNGB Sequence Archive (CNSA) (Guo et al., 2020) of China National GeneBank DataBase (CNGBdb) (Chen et al., 2020) with accession number CNP0002052.
