# Supplementary material for: Chromosome-Scale Genome of Masked Palm Civet (Paguma larvata) Shows Genomic Signatures of Its Biological Characteristics and Evolution
Source: Front Genet. 2022 Jan 21;12:819493. doi: 10.3389/fgene.2021.819493 (PMC8815822; doi:10.3389/fgene.2021.819493)
Supplement: Supplementary file 1 [file DataSheet1.docx]

Supplementary Material

# Supplementary Tables

**Supplementary Table1.** Species information on comparative genomics analysis.

| **Group** | **Species** | **DataBase** | **Genome Version** |
| --- | --- | --- | --- |
| **Omnivores** | *Monodelphis domestica* | Ensembl-100 | ASM229v1 |
|  | *Canis lupusfamiliaris* | Ensembl-100 | CanFam3.1 |
|  | *Pagumalarvata* | CNGB | This study |
|  | *Homo sapiens* | Ensembl-100 | GRCh38 |
|  | *Mus musculus* | Ensembl-100 | GRCm38 |
|  | *Sus scrofa* | Ensembl-100 | Sscrofa11.1 |
| **Herbivores** | *Oryctolagus cuniculus* | Ensembl-100 | OryCun2.0 |
|  | *Bos taurus* | Ensembl-100 | ARS-UCD1.2 |
|  | *Equus caballus* | Ensembl-100 | EquCab3.0 |
|  | *Ailuropoda melanoleuca* | Ensembl-100 | ailMel1 |
|  | *Loxodonta africana* | Ensembl-100 | loxAfr3 |
| **Carnivores** | *Suricata suricatta* | Ensembl-100 | meerkat_22Aug2017_6uvM2_HiC |
|  | *Puma concolor* | NCBI | GCF_003327715.1 |
|  | *Acinonyx jubatus* | NCBI | GCF_003709585.1 |
|  | *Felis catus* | Ensembl-100 | Felis_catus_9.0 |
|  | *Lynx canadensis* | Ensembl-100 | mLynCan4_v1.p |
|  | *Panthera tigrisaltaica* | Ensembl-100 | PanTig1.0 |
|  | *Panthera pardus* | Ensembl-100 | PanPar1.0 |
|  | *Panthera leo* | Ensembl-100 | PanLeo1.0 |
|  | *Ursus maritimus* | Ensembl-100 | UrsMar_1.0 |

**Supplementary Table 2.** Result of BUSCO analysis of the *P. larvata* genome.

| Level | BUSCO assessment results | Dataset | Parameter |
| --- | --- | --- | --- |
| Genome | C:92.1%[S:91.5%,D:0.6%],F:4.4%,M:3.5%,n:4104 | mammalia_odb9 | -m genome |
| Gene | C:92.7%[S:91.9%,D:0.8%],F:2.5%,M:4.8%,n:4104 | mammalia_odb9 | -m proteins |

**Supplementary Table 3.** Statistics of Repeats in the *P. larvata* genome.

| **Type** | **Length (bp)** | **% of genome** |
| --- | --- | --- |
| Trf | 30,320,462 | 1.263899 |
| Repeatmasker | 465,892,433 | 19.42058 |
| Proteinmask | 179,351,348 | 7.476,203 |
| Denovo | 634,379,394 | 26.4439 |
| Total | 782,977,797 | 32.63818 |

**Supplementary Table 4.** Statistics of identified Repeats by *De novo* method in *P. larvata* genome.

| **Type** | **Length (bp)** | **% of genome** |
| --- | --- | --- |
| DNA | 10,857,926 | 0.452609 |
| LINE | 570,167,017 | 23.767229 |
| SINE | 843,585 | 0.035165 |
| LTR | 94,688,316 | 3.947052 |
| Satellite | 458,204 | 0.0191 |
| Simple_repeat | 381,334 | 0.015896 |
| Unknown | 5,414,185 | 0.225689 |
| Total | 634,379,394 | 26.443902 |

**Supplementary Table 5.** Transposable elements in the *P. larvata* genome assembly.

|  | **Repbase TEs** | | **TE proteins** | | ***De novo*** | | **Combined TEs** | |
| --- | --- | --- | --- | --- | --- | --- | --- | --- |
| **Type** | Length (bp) | % in genome | Length (bp) | % in genome | Length (bp) | % in genome | Length (bp) | % in genome |
| **DNA** | 36,300,183 | 1.513161 | 3,240,460 | 0.135078 | 10,857,926 | 0.452609 | 39,983,615 | 1.666704 |
| **LINE** | 341,339,952 | 14.228646 | 169,347,411 | 7.059193 | 570,167,017 | 23.767229 | 656,429,629 | 27.363059 |
| **SINE** | 12,859,164 | 0.53603 | 0 | 0 | 843,585 | 0.035165 | 13,191,480 | 0.549883 |
| **LTR** | 76,803,013 | 3.201509 | 6,781,709 | 0.282693 | 94,688,316 | 3.947052 | 156,158,144 | 6.509402 |
| **Other** | 961 | 0.00004 | 240 | 0.00001 | 0 | 0 | 1,201 | 0.00005 |
| **Unknown** | 0 | 0 | 0 | 0 | 5,414,185 | 0.225689 | 5,414,185 | 0.225689 |
| **Total** | 465,892,433 | 19.420577 | 179,351,348 | 7.476203 | 633,539,856 | 26.408906 | 770,252,760 | 32.107739 |

Note: Repbase TEs: the result of RepeatMasker based on Repbase; TE proteins: the result of RepeatProteinMask based on Repbase; *De novo*: *de novo* finding repeats (Repeatmodeler and LTR_FINDING); Combined TEs: the results obtained from combining the results using all the approaches.

**Supplementary Table 6.** Comparison of protein coding genes between *P. larvata* and other species.

|  | *C. lupus familiaris* | *F. catus* | *H. sapiens* | *L. canadensis* | *M. musculus* | *P. larvata* |
| --- | --- | --- | --- | --- | --- | --- |
| Average CDs length | 1687.60 | 1695.61 | 1715.96 | 1619.43 | 1614.84 | 1585.49 |
| Average exon length | 173.46 | 175.87 | 174.38 | 170.91 | 179.46 | 182.84 |
| Average exon number | 9.73 | 9.64 | 9.84 | 9.48 | 9.00 | 8.67 |
| Average intron length | 4763.13 | 5170.13 | 5516.40 | 5050.65 | 4516.62 | 5095.35 |
| Average mRNA length | 43264.51 | 46372.92 | 50482.98 | 44425.33 | 37741.14 | 40673.75 |
| Total number of exons | 184,178 | 188,625 | 198,677 | 181,187 | 200,901 | 159,024 |
| Total number of genes | 18,931 | 19,564 | 20,190 | 19,122 | 22,326 | 18,339 |
| Total number of introns | 165,247 | 169,061 | 178,487 | 162,065 | 178,575 | 140,685 |
| Average intron number | 8.73 | 8.64 | 8.84 | 8.48 | 8 | 7.67 |

**Supplementary Table 7.** Statistics on functional annotation of the *P. larvata* gene set.

| **Values** | **Total genes** | **Swissprot-Annotated** | **KEGG-Annotated** | **TrEMBL-Annotated** | **Interpro-Annotated** | **GO-Annotated** | **Overall annotated genes** |
| --- | --- | --- | --- | --- | --- | --- | --- |
| **Number** | 18,340 | 18,116 | 16,621 | 18,328 | 18,080 | 12,898 | 18,333 |
| **Percentage** | 100% | 98.78% | 90.63% | 99.93% | 98.58% | 70.33% | 99.96% |

**Supplementary Table 8.** Statistics of ncRNA annotation.

| **Type** |  | **number** | **Average length(bp)** | **Total length(bp)** | **% of genome** |
| --- | --- | --- | --- | --- | --- |
| **miRNA** | | 1097 | 78.03 | 85,599 | 0.0035 |
| **tRNA** | | 45,835 | 83.88 | 3,844,815 | 0.16 |
| **rRNA** | rRNA | 714 | 76.35 | 54,513 | 0.0023 |
|  | 18S | 28 | 196.64 | 5506 | 0.00023 |
|  | 28S | 91 | 107.50 | 9783 | 0.00041 |
|  | 5.8S | 1 | 78.00 | 78 | 0.0000030 |
|  | 5S | 594 | 65.90 | 39,146 | 0.0016 |
| **snRNA** | snRNA | 1942 | 115.06 | 223,446 | 0.0093 |
|  | CD-box | 321 | 90.92 | 29,186 | 0.00122 |
|  | HACA-box | 267 | 137.40 | 36,683 | 0.0015 |
|  | splicing | 1318 | 115.50 | 152,235 | 0.0063 |

**Supplementary Table 9.** Anchoring genes in the chromosome X of *H. sapiens*, *F. catus* and *S. suricatta* to the *P. larvata* genome.

|  | *H. sapiens* | *F. catus* | *S. suricatta* |
| --- | --- | --- | --- |
| Gene Number in chromosome X | 847 | 796 | 737 |
| Mapped to *P. larvata* genome | 800 | 795 | 735 |
| Percentage (%) | 94.45 | 99.87 | 99.73 |
| Anchored to Chr22 of *P. larvata* | 726 | 753 | 676 |
| Percentage (%) | 85.71 | 94.60 | 91.72 |

**Supplementary Table 10.** Sequencing depth of sex-link regions in 45 individuals.

| **Sample** | **Sex** | **Chr22** | **Scaf457** | **Autosomes** |
| --- | --- | --- | --- | --- |
| **Civet_wgs_1** | Female | 22.79 | 6.26 | 21.46 |
| **Civet_wgs_2** | Female | 21.90 | 5.19 | 21.39 |
| **Civet_wgs_3** | Female | 13.59 | 3.82 | 13.87 |
| **Civet_wgs_4** | Male | 10.05 | 10.28 | 16.51 |
| **Civet_wgs_5** | Male | 13.81 | 13.85 | 21.78 |
| **Civet_wgs_6** | Female | 23.34 | 5.71 | 22.24 |
| **Civet_wgs_7** | Female | 21.33 | 5.61 | 20.46 |
| **Civet_wgs_8** | Male | 12.25 | 12.07 | 20.36 |
| **Civet_wgs_9** | Female | 17.19 | 4.40 | 16.65 |
| **Civet_wgs_10** | Female | 24.15 | 5.00 | 23.24 |
| **Civet_wgs_11** | Female | 19.86 | 4.07 | 19.68 |
| **Civet_wgs_12** | Male | 14.69 | 15.03 | 22.23 |
| **Civet_wgs_13** | Female | 17.82 | 4.22 | 17.64 |
| **Civet_wgs_14** | Male | 14.58 | 15.52 | 23.93 |
| **Civet_wgs_15** | Male | 14.66 | 15.04 | 23.46 |
| **Civet_wgs_16** | Male | 12.30 | 13.11 | 20.19 |
| **Civet_wgs_17** | Female | 21.60 | 5.64 | 20.69 |
| **Civet_wgs_18** | Male | 12.13 | 12.97 | 19.85 |
| **Civet_wgs_19** | Female | 22.22 | 6.46 | 21.38 |
| **Civet_wgs_20** | Male | 15.11 | 16.13 | 24.80 |
| **Civet_wgs_21** | Female | 23.23 | 5.77 | 22.72 |
| **Civet_wgs_22** | Female | 25.58 | 6.39 | 24.53 |
| **Civet_wgs_23** | Male | 11.95 | 12.75 | 19.26 |
| **Civet_wgs_24** | Female | 16.08 | 3.37 | 15.70 |
| **Civet_wgs_25** | Male | 14.40 | 15.74 | 23.46 |
| **Civet_wgs_26** | Female | 27.33 | 5.53 | 25.68 |
| **Civet_wgs_27** | Female | 17.34 | 4.19 | 16.94 |
| **Civet_wgs_28** | Male | 13.74 | 14.57 | 22.08 |
| **Civet_wgs_29** | Female | 21.98 | 6.11 | 21.08 |
| **Civet_wgs_30** | Male | 12.12 | 13.52 | 19.67 |
| **Civet_51** | Male | 12.58 | 13.03 | 20.80 |
| **Civet_52** | Female | 19.96 | 4.69 | 19.28 |
| **Civet_53** | Female | 19.49 | 5.16 | 19.15 |
| **Civet_54** | Male | 13.25 | 13.73 | 21.67 |
| **Civet_55** | Male | 17.36 | 18.00 | 28.51 |
| **Civet_56** | Female | 28.08 | 7.59 | 26.62 |
| **Civet_57** | Male | 15.59 | 16.64 | 24.89 |
| **Civet_58** | Female | 21.71 | 5.71 | 21.21 |
| **Civet_59** | Male | 13.34 | 13.82 | 21.48 |
| **Civet_60** | Female | 16.55 | 5.16 | 18.08 |
| **Civet_61** | Female | 23.3 | 5.92 | 22.41 |
| **Civet_62** | Female | 24.92 | 7.54 | 23.67 |
| **Civet_63** | Female | 20.69 | 5.49 | 20.21 |
| **Civet_64** | Female | 21.69 | 6.15 | 21.24 |
| **Civet_65** | Female | 21.44 | 5.74 | 20.55 |

**Supplementary Table 24.** Gene expression (TPM) of the *ACE2*, *TMPRSS2* and *TMPRSS4* in16 organs*.*

| Sample | Organ | *ACE2* | *TMPRSS2* | *TMPRSS4* |
| --- | --- | --- | --- | --- |
| heart1 | heart | 0.62 | 0.05 | 0 |
| heart2 | heart | 1.30 | 0.03 | 0 |
| heart3 | heart | 0.73 | 0.03 | 0 |
| lung1 | lung | 8.17 | 60.17 | 6.98 |
| lung2 | lung | 7.25 | 62.81 | 6.38 |
| lung3 | lung | 8.62 | 56.83 | 6.71 |
| spleen1 | spleen | 0.53 | 0 | 0.06 |
| spleen2 | spleen | 0.41 | 0.03 | 0 |
| spleen3 | spleen | 0.46 | 0 | 0 |
| liver1 | liver | 5.53 | 0.39 | 0 |
| liver2 | liver | 5.25 | 0.38 | 0 |
| liver3 | liver | 5.34 | 0.3 | 0 |
| kidney1 | kidney | 88.74 | 61.23 | 0.09 |
| kidney2 | kidney | 85.35 | 62.84 | 0 |
| kidney3 | kidney | 77.96 | 71.76 | 0.06 |
| esophagus1 | esophagus | 24.28 | 22.01 | 4.52 |
| esophagus2 | esophagus | 23.24 | 18.81 | 4.44 |
| esophagus3 | esophagus | 23.39 | 20.12 | 4.30 |
| stomach1 | stomach | 1.15 | 97.71 | 0 |
| stomach2 | stomach | 1.21 | 94.43 | 0 |
| stomach3 | stomach | 2.01 | 99.03 | 0 |
| colon1 | colon | 663.48 | 69.67 | 47.70 |
| colon2 | colon | 646.14 | 68.06 | 47.68 |
| colon3 | colon | 644.33 | 70.34 | 49.40 |
| rectum1 | rectum | 635.17 | 94.01 | 57.59 |
| rectum2 | rectum | 627.34 | 92.81 | 56.53 |
| rectum3 | rectum | 614.26 | 99.96 | 58.65 |
| cecum1 | cecum | 618.17 | 84.54 | 48.77 |
| cecum2 | cecum | 582.7 | 85.94 | 46.65 |
| cecum3 | cecum | 519.48 | 81.69 | 48.88 |
| duodenum1 | duodenum | 245.72 | 15.72 | 8.99 |
| duodenum2 | duodenum | 226.05 | 17.84 | 8.84 |
| duodenum3 | duodenum | 307.58 | 20.17 | 11.76 |
| jejunum1 | jejunum | 111.02 | 7.03 | 8.07 |
| jejunum2 | jejunum | 252.01 | 14.06 | 10.28 |
| jejunum3 | jejunum | 301.02 | 17.96 | 11.52 |
| testis1 | testis | 0.29 | 54.33 | 0 |
| testis2 | testis | 0.51 | 58.57 | 0 |
| testis3 | testis | 0.51 | 58.49 | 0.02 |
| vas_deferens1 | vas deferens | 0.91 | 1.55 | 0.03 |
| vas_deferens2 | vas deferens | 1.4 | 2.04 | 0.08 |
| vas_deferens3 | vas deferens | 1.09 | 2.14 | 0.12 |
| bladder1 | bladder | 55.87 | 46.80 | 0.37 |
| bladder2 | bladder | 52.75 | 56.97 | 0.32 |
| bladder3 | bladder | 29.80 | 40.14 | 0.35 |
| spinal1 | spinal cord | 0.34 | 0.03 | 0.15 |
| spinal2 | spinal cord | 0.4 | 0.09 | 0.32 |
| spinal3 | spinal cord | 0.3 | 0.03 | 0.37 |

**Supplementary Table 25.** The genome-wide heterozygosity in *P. larvata* and 36 published mammals.

| Species | Heterozygosity (%) | Sources |
| --- | --- | --- |
| Iberian lynx (*Lynx pardinus*) | 0.010 | Abascal et al. 2016(Abascal et al., 2016) |
| Cheetah (*Acinonyx jubatus*) | 0.020 | Dobrynin et al. 2015(Dobrynin et al., 2015) |
| Snow leopard (*Panthera uncia syn*) | 0.023 | Cho et al. 2013(Cho et al., 2013) |
| Yangtze river dolphin (*Lipotes vexillifer*) | 0.026 | Zhou et al. 2013(Zhou et al., 2013) |
| Siberian tiger (*Panthera tigris altaica)* | 0.030 | Dobrynin et al. 2015(Dobrynin et al., 2015) |
| Domestic dog (*Canis familiaris*) | 0.032 | Lindblad-Toh et al. 2005(Lindblad-Toh et al., 2005) |
| Bengal tiger (*Panthera tigris tigris*) | 0.040 | Dobrynin et al. 2015(Dobrynin et al., 2015) |
| Domestic Turkey (*Meleagris gallopavo*) | 0.057 | Dalloul et al. 2010(Dalloul et al., 2010) |
| African lion (*Panthera leo*) | 0.058 | Cho et al. 2013(Cho et al., 2013) |
| Pileated gibbon (*Hylobates pileatus*) | 0.073 | Carbone et al.2014(Carbone et al., 2014) |
| Platypus (*Ornithorhynchus anatinus*) | 0.075 | Warren et al. 2008(Warren et al., 2008) |
| Human_Han (*Homo species*) | 0.077 | Meyer et al.2012(Meyer et al., 2012) |
| Finless porpoise (*Neophocaena phocaenoides*) | 0.086 | Yim et al. Nat. 2014(Yim et al., 2014) |
| Tibetan antelope (*Pantholops hodgsonii*) | 0.088 | Ge et al. Nat. 2013(Ge et al., 2013) |
| Yak (*Bos grunniens*) | 0.089 | Qiu et al. Nat. 2012(Qiu et al., 2012) |
| Southern white rhinoceros (*Ceratotherium simum simum*) | 0.090 | Tunstall et al. 2018(Tunstall et al., 2018) |
| Common chimpanzee (*Pan troglodytes*) | 0.095 | Mikkelsen et al. 2005(Mikkelsen et al., 2005) |
| Domestic horse (*Equus caballus*) | 0.095 | Wade et al. 2009(Wade et al., 2009) |
| Polar bear (*Ursus maritimus*) | 0.108 | Liu et al. 2014(Liu et al., 2014) |
| Brown hyena (*Parahyaena brunnea*) | 0.121 | Westbury et al. 2018(Westbury et al., 2018) |
| Oimyakon wooly mammoth (*Mammuthus primigenius*) | 0.125 | Palkopoulou et al. 2016(Palkopoulou et al., 2015) |
| Rat (*Rattus norvegicus*) | 0.125 | Leffler et al.2012(Leffler et al., 2012) |
| Giant panda (*Ailuropoda melanoleuca*) | 0.132 | Li et al. 2010(Li et al., 2010) |
| Gray wolf (*Canis lupus*) | 0.149 | Corbett-Detig et al. 2015(Corbett-Detig et al., 2015) |
| Chinese hamster (*Cricetulus griseus*) | 0.159 | Lewis et al. 2013(Lewis et al., 2013) |
| Rhesus macaque (*Macaca mulatta*) | 0.287 | Corbett-Detig et al. 2015(Corbett-Detig et al., 2015) |
| Brown bear (*Ursus arctos*) | 0.320 | Liu et al. 2014(Liu et al., 2014) |
| Common marmoset (*Callithrix jacchus*) | 0.341 | Worley et al. 2014(Sequencing and Consortium, 2014) |
| Przewalski's horse (*Equus ferus przewalskii*) | 0.363 | Corbett-Detig et al. 2015(Corbett-Detig et al., 2015) |
| Brandt’s bat (*Myotis brandtii*) | 0.371 | Seim et al. 2013(Seim et al., 2013) |
| Chinese rhesus macaque (*Macaca mulatta lasiota*) | 0.410 | Yan et al. 2011(Yan et al., 2011) |
| Wild boar (*Sus scrofa*) | 0.441 | Corbett-Detig et al. 2015(Corbett-Detig et al., 2015) |
| Masked palm civet (*Paguma larvata*) | 0.4726 | This study |
| Opossum (*Monodelphis domestica*) | 0.490 | Mikkelsen et al. 2007(Mikkelsen et al., 2007) |
| Crab-eating macaque (*Macaca fascicularis*) | 0.530 | Yan et al. 2011(Yan et al., 2011) |
| Rabbit (*Oryctolagus cuniculus*) | 0.750 | Carneiro et al. 2014(Carneiro et al., 2014) |
| House mouse (*Mus musculus castaneus*) | 0.809 | Corbett-Detig et al.2015(Corbett-Detig et al., 2015) |

**Reference:**

Abascal, F., Corvelo, A., Cruz, F., Villanueva-Cañas, J.L., Vlasova, A., Marcet-Houben, M., et al. (2016). Extreme genomic erosion after recurrent demographic bottlenecks in the highly endangered Iberian lynx. *Genome biology* 17(1)**,** 1-19.

Carbone, L., Harris, R.A., Gnerre, S., Veeramah, K.R., Lorente-Galdos, B., Huddleston, J., et al. (2014). Gibbon genome and the fast karyotype evolution of small apes. *Nature* 513(7517)**,** 195-201.

Carneiro, M., Rubin, C.-J., Di Palma, F., Albert, F.W., Alföldi, J., Barrio, A.M., et al. (2014). Rabbit genome analysis reveals a polygenic basis for phenotypic change during domestication. *Science* 345(6200)**,** 1074-1079.

Cho, Y.S., Hu, L., Hou, H., Lee, H., Xu, J., Kwon, S., et al. (2013). The tiger genome and comparative analysis with lion and snow leopard genomes. *Nature communications* 4(1)**,** 1-7.

Corbett-Detig, R.B., Hartl, D.L., and Sackton, T.B. (2015). Natural selection constrains neutral diversity across a wide range of species. *PLoS biology* 13(4)**,** e1002112.

Dalloul, R.A., Long, J.A., Zimin, A.V., Aslam, L., Beal, K., Ann Blomberg, L., et al. (2010). Multi-platform next-generation sequencing of the domestic turkey (Meleagris gallopavo): genome assembly and analysis. *PLoS biology* 8(9)**,** e1000475.

Dobrynin, P., Liu, S., Tamazian, G., Xiong, Z., Yurchenko, A.A., Krasheninnikova, K., et al. (2015). Genomic legacy of the African cheetah, Acinonyx jubatus. *Genome biology* 16(1)**,** 1-20.

Ge, R.-L., Cai, Q., Shen, Y.-Y., San, A., Ma, L., Zhang, Y., et al. (2013). Draft genome sequence of the Tibetan antelope. *Nature communications* 4(1)**,** 1-7.

Leffler, E.M., Bullaughey, K., Matute, D.R., Meyer, W.K., Segurel, L., Venkat, A., et al. (2012). Revisiting an old riddle: what determines genetic diversity levels within species?

Lewis, N.E., Liu, X., Li, Y., Nagarajan, H., Yerganian, G., O'brien, E., et al. (2013). Genomic landscapes of Chinese hamster ovary cell lines as revealed by the Cricetulus griseus draft genome. *Nature biotechnology* 31(8)**,** 759-765.

Li, R., Fan, W., Tian, G., Zhu, H., He, L., Cai, J., et al. (2010). The sequence and de novo assembly of the giant panda genome. *Nature* 463(7279)**,** 311-317.

Lindblad-Toh, K., Wade, C.M., Mikkelsen, T.S., Karlsson, E.K., Jaffe, D.B., Kamal, M., et al. (2005). Genome sequence, comparative analysis and haplotype structure of the domestic dog. *Nature* 438(7069)**,** 803-819.

Liu, S., Lorenzen, E.D., Fumagalli, M., Li, B., Harris, K., Xiong, Z., et al. (2014). Population genomics reveal recent speciation and rapid evolutionary adaptation in polar bears. *Cell* 157(4)**,** 785-794.

Meyer, M., Kircher, M., Gansauge, M.-T., Li, H., Racimo, F., Mallick, S., et al. (2012). A high-coverage genome sequence from an archaic Denisovan individual. *Science* 338(6104)**,** 222-226.

Mikkelsen, T., Hillier, L., Eichler, E., Zody, M., Jaffe, D., Yang, S.-P., et al. (2005). Initial sequence of the chimpanzee genome and comparison with the human genome. *Nature* 437(7055)**,** 69-87.

Mikkelsen, T.S., Wakefield, M.J., Aken, B., Amemiya, C.T., Chang, J.L., Duke, S., et al. (2007). Genome of the marsupial Monodelphis domestica reveals innovation in non-coding sequences. *Nature* 447(7141)**,** 167-177.

Palkopoulou, E., Mallick, S., Skoglund, P., Enk, J., Rohland, N., Li, H., et al. (2015). Complete genomes reveal signatures of demographic and genetic declines in the woolly mammoth. *Current Biology* 25(10)**,** 1395-1400.

Qiu, Q., Zhang, G., Ma, T., Qian, W., Wang, J., Ye, Z., et al. (2012). The yak genome and adaptation to life at high altitude. *Nature genetics* 44(8)**,** 946-949.

Seim, I., Fang, X., Xiong, Z., Lobanov, A.V., Huang, Z., Ma, S., et al. (2013). Genome analysis reveals insights into physiology and longevity of the Brandt’s bat Myotis brandtii. *Nature communications* 4(1)**,** 1-8.

Sequencing, T.M.G., and Consortium, A. (2014). The common marmoset genome provides insight into primate biology and evolution. *Nature genetics* 46(8)**,** 850.

Tunstall, T., Kock, R., Vahala, J., Diekhans, M., Fiddes, I., Armstrong, J., et al. (2018). Evaluating recovery potential of the northern white rhinoceros from cryopreserved somatic cells. *Genome research* 28(6)**,** 780-788.

Wade, C., Giulotto, E., Sigurdsson, S., Zoli, M., Gnerre, S., Imsland, F., et al. (2009). Genome sequence, comparative analysis, and population genetics of the domestic horse. *Science* 326(5954)**,** 865-867.

Warren, W.C., Hillier, L.W., Graves, J.A.M., Birney, E., Ponting, C.P., Grützner, F., et al. (2008). Genome analysis of the platypus reveals unique signatures of evolution. *Nature* 453(7192)**,** 175.

Westbury, M.V., Hartmann, S., Barlow, A., Wiesel, I., Leo, V., Welch, R., et al. (2018). Extended and continuous decline in effective population size results in low genomic diversity in the world’s rarest hyena species, the brown hyena. *Molecular biology and evolution* 35(5)**,** 1225-1237.

Yan, G., Zhang, G., Fang, X., Zhang, Y., Li, C., Ling, F., et al. (2011). Genome sequencing and comparison of two nonhuman primate animal models, the cynomolgus and Chinese rhesus macaques. *Nature biotechnology* 29(11)**,** 1019-1023.

Yim, H.-S., Cho, Y.S., Guang, X., Kang, S.G., Jeong, J.-Y., Cha, S.-S., et al. (2014). Minke whale genome and aquatic adaptation in cetaceans. *Nature genetics* 46(1)**,** 88-92.

Zhou, X., Sun, F., Xu, S., Fan, G., Zhu, K., Liu, X., et al. (2013). Baiji genomes reveal low genetic variability and new insights into secondary aquatic adaptations. *Nature communications* 4(1)**,** 1-6.

# Supplementary Figures


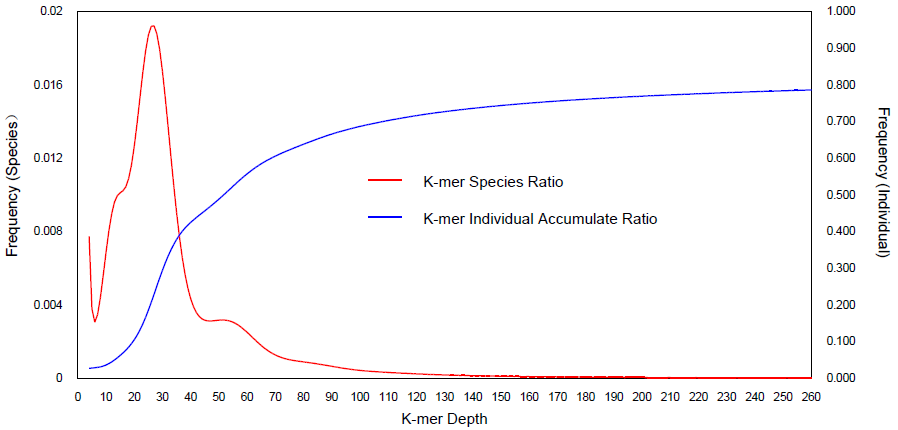


**Supplementary Figure 1.** Estimation of the genome size of *P. larvata* by K-mer analysis. The frequency and sequencing depth of 17 k-mer were plotted. The red line represents the ratio of k-mer species with difference depths, while the blue line represents the accumulated ratio of individual k-mers.


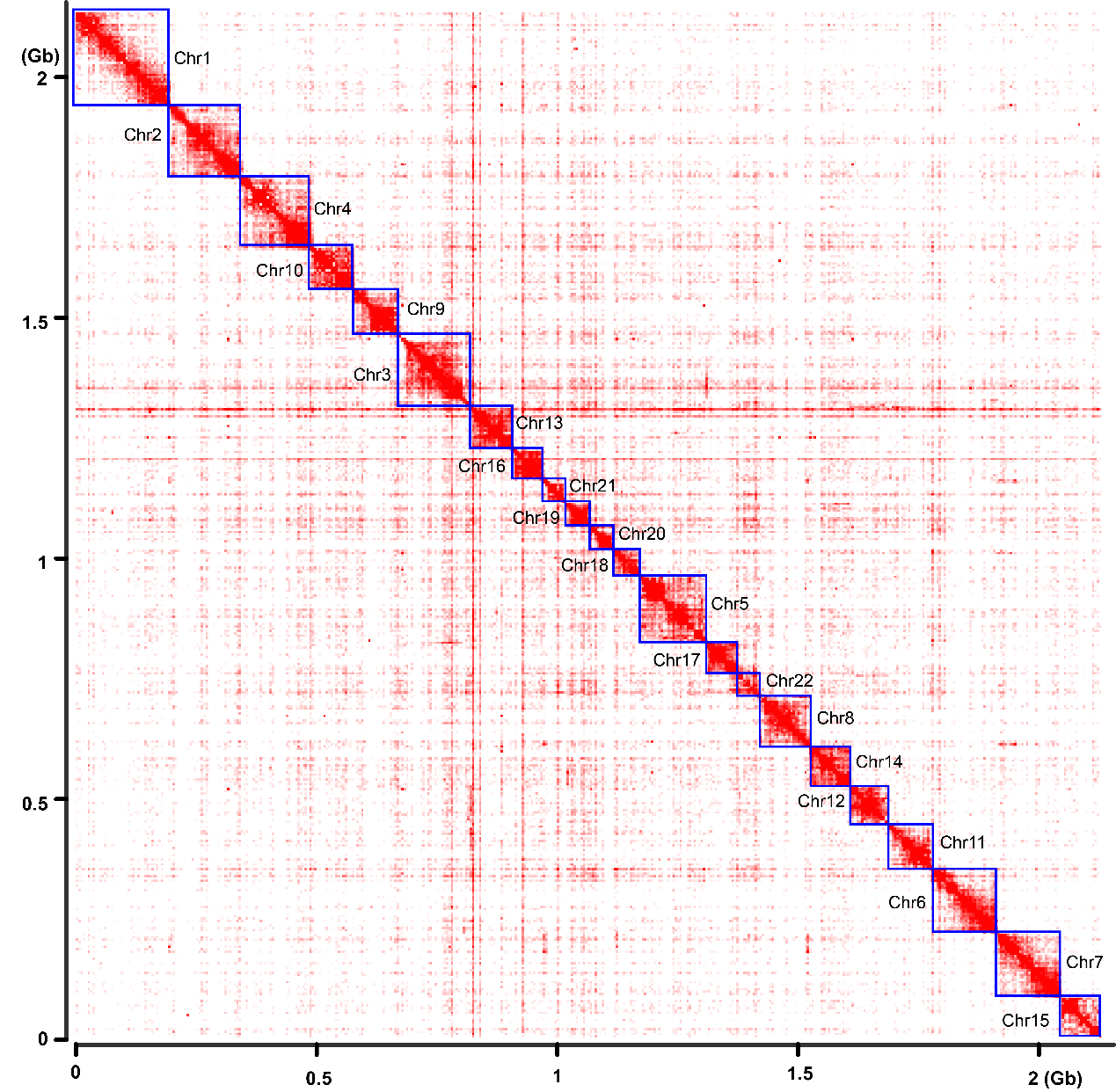


**Supplementary Figure 2.** Contig contact matrix of our assembled *P. larvata* genome. The color depth represents the density of Hi-C interactions.


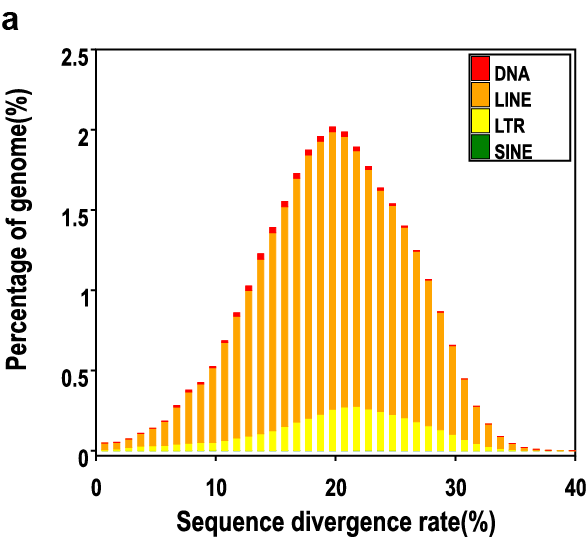

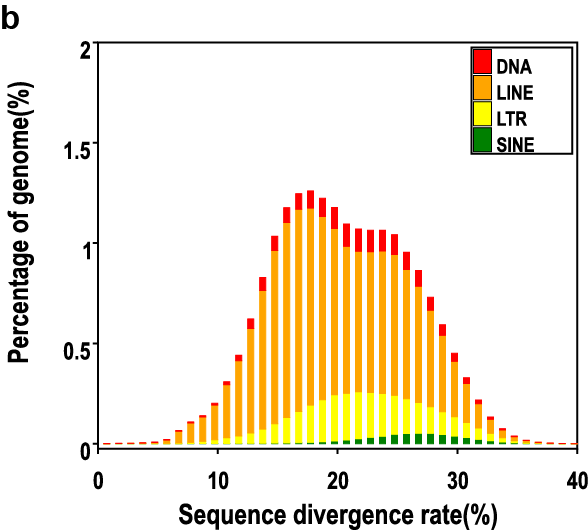


**Supplementary Figure 3.** Distribution of divergence rate of each type of *P. larvata's* TE. a) The divergence rate was calculated between the identified TE elements in the genome by *De novo*method and the consensus sequence in the predicted TE library. b) The divergence rate was calculated between the identified TE elements in the genome by homology-based method and the consensus sequence in the Repbase database.

**
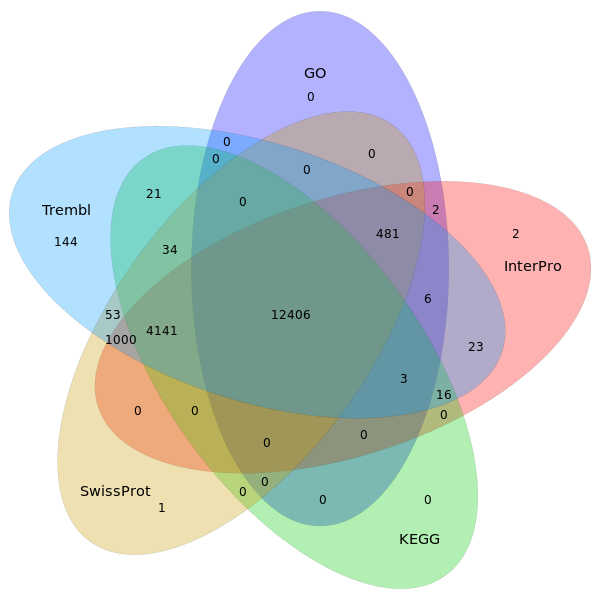
**

**Supplementary Figure 4.** Statistics of functional annotation of *P. larvata* gene set.

**
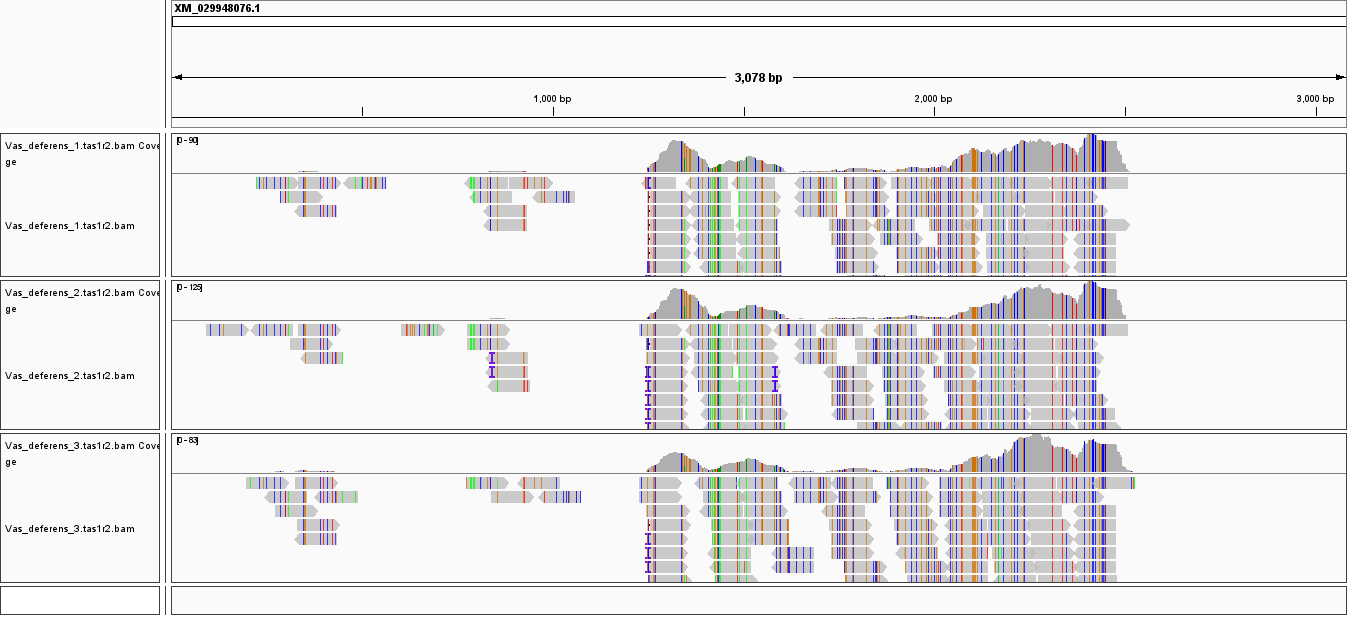
**

**Supplementary Figure 5.** The coverage distribution of RNA-seq data mapped to the *Tas1r2* gene of the *S. suricatta* (XM_029948076.1)*.*


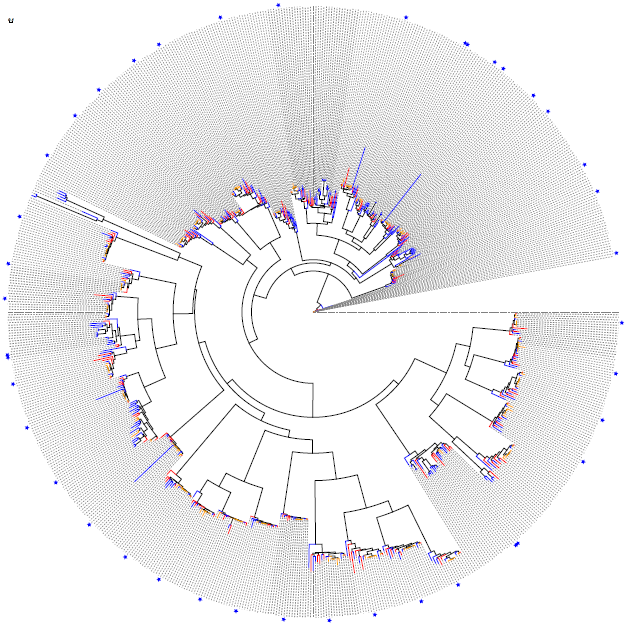


**Supplementary Figure 6.** The phylogenetic tree of CYP450 gene family.Blue star represents species*P. larvata*, and the red,blue, and orange branch colors represent herbivorous, omnivorous, and carnivorous, respectively.


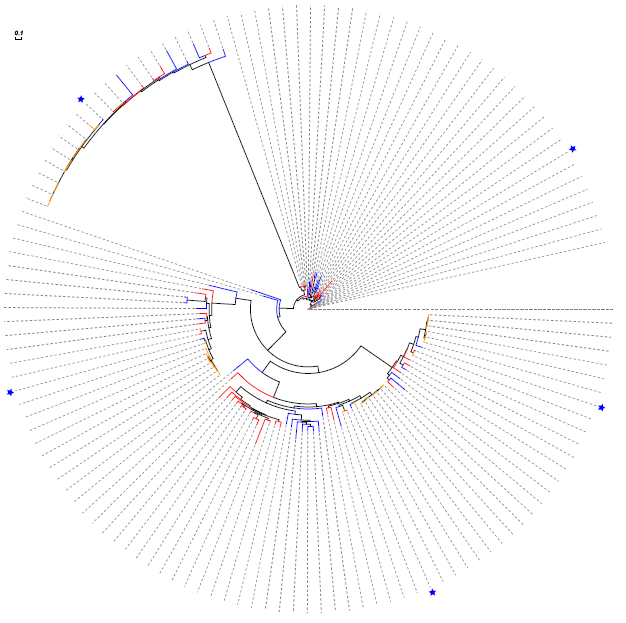


**Supplementary Figure 7.** The phylogenetic tree of CES gene family.Blue star represents species*P. larvata*, and the red,blue, and orange branch colors represent herbivorous, omnivorous, and carnivorous, respectively.


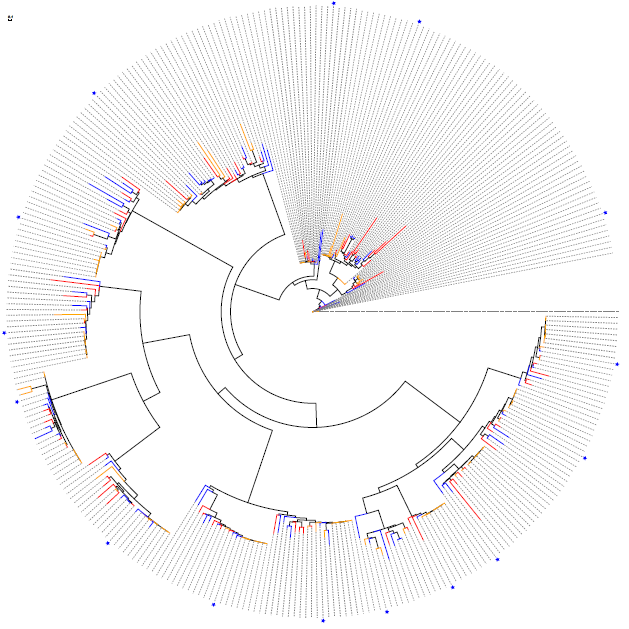


**Supplementary Figure 8.** The phylogenetic tree of GST gene family.Blue star represents species*P. larvata*, and the red,blue, and orange branch colors represent herbivorous, omnivorous, and carnivorous, respectively.


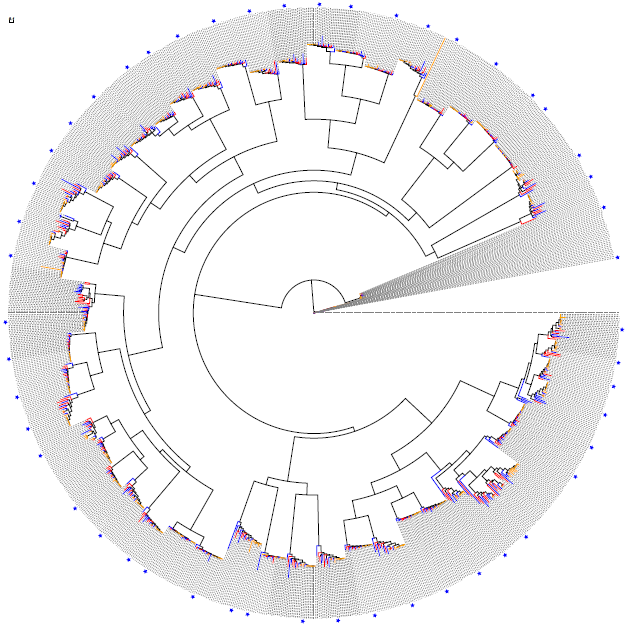


**Supplementary Figure 9.** The phylogenetic tree of ABC gene family.Blue star represents species*P. larvata*, and the red,blue, and orange branch colors represent herbivorous, omnivorous, and carnivorous, respectively.


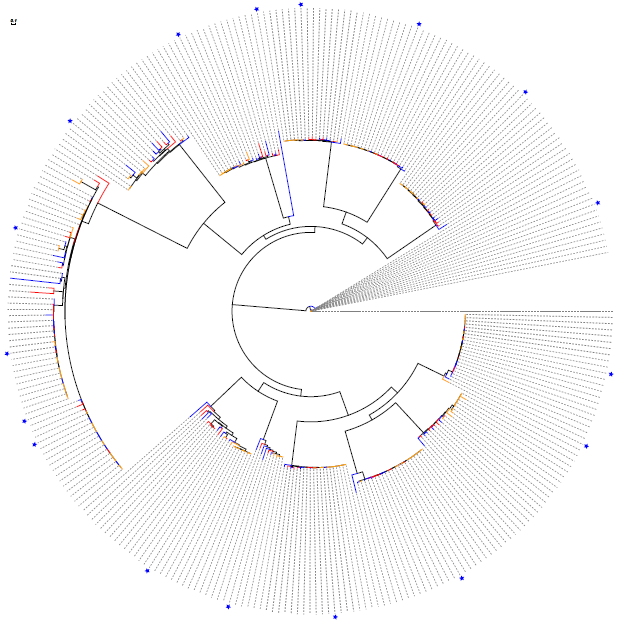


**Supplementary Figure 10.** The phylogenetic tree of UGT gene family.Blue star represents species*P. larvata*, and the red,blue, and orange branch colors represent herbivorous, omnivorous, and carnivorous, respectively.


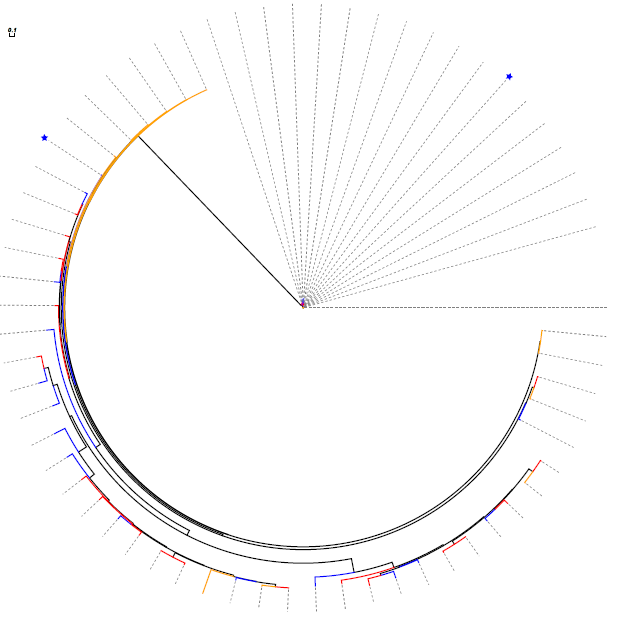


**Supplementary Figure 11.** The phylogenetic tree of AOX gene family.Blue star represents species*P. larvata*, and the red,blue, and orange branch colors represent herbivorous, omnivorous, and carnivorous, respectively.


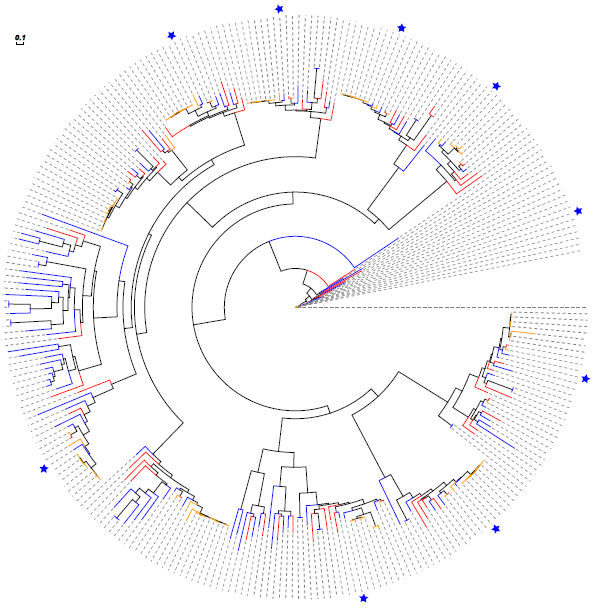


**Supplementary Figure 12.** The phylogenetic tree of TAS2R gene family. Blue star represents species*P. larvata*, and the red,blue, and orange branch colors represent herbivorous, omnivorous, and carnivorous, respectively.


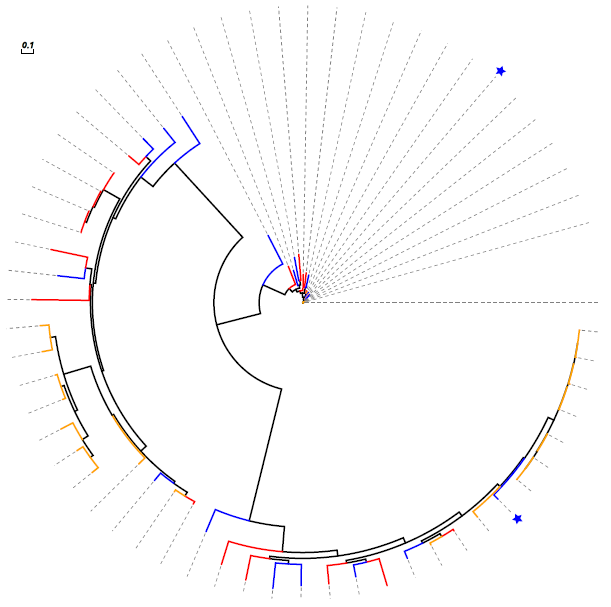


**Supplementary Figure 13.** The phylogenetic tree of TAS1R gene family. Blue star represents species *P. larvata*, and the red, blue, and orange branch colors represent herbivorous, omnivorous, and carnivorous, respectively.


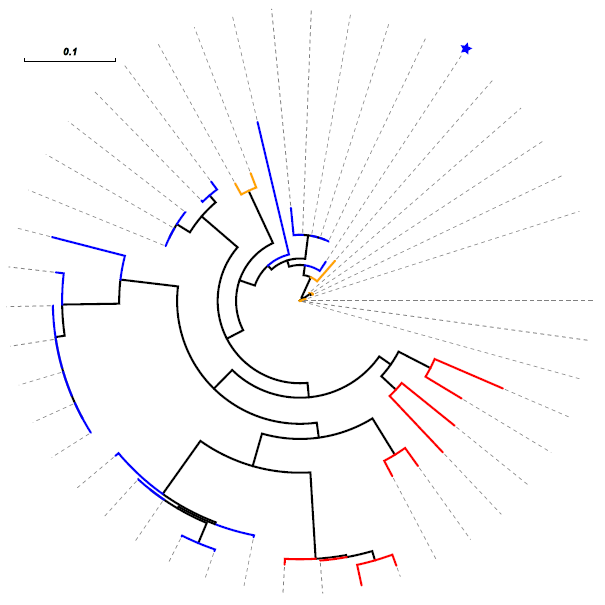


**Supplementary Figure 14.** The phylogenetic tree of AMY gene family. Blue star represents species *P. larvata*, and the red, blue, and orange branch colors represent herbivorous, omnivorous, and carnivorous, respectively.

# Scripts and commands

**3.1 Assembly of primary genome using stLFR data:**

supernova run --id=$PROJECT_NAME --maxreads=$MAX_READS --fastqs=./ --localcores=$THREADS --localmem=$MEMORY --nopreflight --accept-extreme-coverage >supernova_run.log 2>supernova_run.err || exit 1

supernova mkoutput --minsize=$MINSIZE --style=pseudohap --asmdir="./"$PROJECT_NAME"/outs/assembly" --outprefix="$PROJECT_NAME""_output" >supernova_mkoutput.log 2>supernova_mkoutput.err || exit 1

**3.2 Hi-C assembly**

juicer.sh -t 16 -z genome.fa -p sizes -y MboI.txt -s MboI -S early -d ./ -D juicer/CPU

3d-dna-master/run-asm-pipeline.sh genome.fa merged_nodups.txt

**3.3 Repeat annotation**

ltr_finder -w 2 -s Athal-tRNAs.fagenome.fa. 1>ltr_finder 2>>log

RepeatMasker -nolow -no_is -norna -engine ncbi -parallel 1 -lib library genome.fa> log 2> log2

RepeatModeler -engine ncbi -database mydb -pa 9 >run.out

RepeatProteinMask -engine ncbi -noLowSimple -pvalue 0.0001 genome.fa

trfgenome.fa 2 7 7 80 10 50 2000 -d -h

**3.4 Gene annotation**

genewise -trev -genesf -sum -gff ./ass.cut.2k.fa ./Target.pep > ./Target_ass.cut.2k.genewise

spaln -O0 -Q7 -pw -T felicatu -t12 -M1 -dPlar Target.pep.gz > Target.spaln.gff3 2>Target.spaln.log

stringtie ./merged_sorted.bam -p 1 -o ./transcripts.gtf

maker maker_opts.ctl maker_bopts.ctl maker_exe.ctl --cpus 1 -RM_off --ignore_nfs_tmp

**3.5 Functional annotation**

interproscan-5.52-86.0/interproscan.sh -goterms -dp -f tsv -T temp -ipep -o pep.iprscan;

blastall -p blastp -e 1e-05 -a 2 -m 8 -F F -d database -i pep -o result

**3.5 Gene expansion and contraction**

tree (MDOM:159,(LAFR:103,((HSAP:87,(MMUS:80,OCUN:80):7):6,(((SSCR:62,BTAU:62):18,ECAB:80):1,(((AMEL:19,UMAR:19):26,CLUP:45):10,((PLAR:34,SSUR:34):4,(((PCON:9,AJUB:9):2,(FCAT:10,LCAN:10):1):3,(PTIG:5,(PPAR:4,PLEO:4):1):9):25):16):26):12):10):56)

load -i ./in.tab -p 0.05 -t 12 -r 10000 -l ./global.log

lambda -s

report ./global.out

**3.6 Construction of phylogenetic tree**

iqtree -s all.phy -bb 1000 -nt 12 -alrt 1000

**3.7 Variants calling and Quality control**

Sentieon driver -r genome.fa -t 48 --algo GVCFtyper -v sample1.g.vcf.gz vcf.gz

gatk --java-options "-Xmx5g" SelectVariants --select-type-to-include SNP -R genome.fa -V vcf.gz -O SNP.vcf.gz ; gatk --java-options "-Xmx5g" SelectVariants --select-type-to-include INDEL -R genome.fa -V vcf.gz -O indel.vcf.gz

gatk --java-options "-Xmx50g" VariantFiltration -V SNP.vcf.gz --filter-expression "QD < 2.0 || FS > 60.0 || MQ < 40.0 || MQRankSum< -12.5 || ReadPosRankSum< -8.0" --filter-name "snp_filter" -O SNP_hardfil.vcf.gz; gatkSelectVariants --exclude-filtered true -R genome.fa -V SNP_hardfil.vcf.gz -O SNP_hardfil_true.vcf.gz

**3.8 PSMC and SMC++ analysis**

Samtools mpileup -C50 -ufgenome.fadeduped.bam | bcftools view -c - | bcftools/vcfutils.pl vcf2fq -d 12 -D 70 >newdepth.fa

fq2psmcfa -q20 newdepth.fa.gz >psmc.fa

splitfa psmc.fa>psmc.split.fa

seq 100 | xargs -i echo psmc -N25 -t15 -r5 -b -p "4+25*2+4+6" -o splitfa.round-{}.psmcpsmc.split.fa | sh

smc++ vcf2smc --cores 1 –mask mask.bed.gz -d samplesample vcf.gz smc.gz genome Sample: all samples

smc++ estimate --cores 24 -o ./ 2.4e-09 smc.gz --knots 21

**3.9 Genetic diversity analysis**

vcftools --het --gzvcf vcf.gz --out vcf.gz.stat

**3.10 Gene expression analysis**

perl /trinityrnaseq/2.11.0/util/align_and_estimate_abundance.pl --transcripts Plar.cds --seqTypefq --left x_1.paired.fq.gz --right x_2.paired.fq.gz --est_method RSEM --aln_method bowtie2 --output_dir result
